# Addressing challenges in the removal of unbound dye from passively labelled extracellular vesicles†

**DOI:** 10.1039/d1na00755f

**Published:** 2021-11-23

**Authors:** Kaisa Rautaniemi, Jacopo Zini, Emilia Löfman, Heikki Saari, Iida Haapalehto, Johanna Laukka, Sami Vesamäki, Alexander Efimov, Marjo Yliperttula, Timo Laaksonen, Elina Vuorimaa-Laukkanen, Ekaterina S. Lisitsyna

**Affiliations:** Chemistry and Advanced Materials, Faculty of Engineering and Natural Sciences, Tampere University Korkeakoulunkatu 8 33720 Tampere Finland ekaterina.lisitsyna@tuni.fi; Drug Research Program, Division of Pharmaceutical Biosciences, Faculty of Pharmacy, University of Helsinki Viikinkaari 5 00790 Helsinki Finland; Finnish Red Cross Blood Services Kivihaantie 7 00310 Helsinki Finland

## Abstract

Studies of extracellular vesicles (EVs), their trafficking and characterization often employ fluorescent labelling. Unfortunately, little attention has been paid thus far to a thorough evaluation of the purification of EVs after labelling, although the presence of an unbound dye may severely compromise the results or even lead to wrong conclusions on EV functionality. Here, we systematically studied five dyes for passive EV labelling and meticulously compared five typical purification methods: ultracentrifugation (UC), ultracentrifugation with discontinuous density gradient (UCG), ultrafiltration (UF), size exclusion chromatography (SEC), and anion exchange chromatography (AEC). A general methodology for evaluation of EV purification efficiency after the labelling was developed and tested to select the purification methods for the chosen dyes. Firstly, we found that some methods initially lead to high EV losses even in the absence of the dye. Secondly, the suitable purification method needs to be found for each particular dye and depends on the physical and chemical properties of the dye. Thirdly, we demonstrated that the developed parameter *E*_rp_ (relative purification efficiency) is a useful tool for the pre-screening of the suitable dye-purification method combinations. Additionally, it was also shown that the labelled EVs properly purified from the unbound dye may show significantly reduced contrast and visibility in the target application, *e.g.* in the live cell fluorescence lifetime imaging.

## Abstract 2

We propose and test a systematic approach for choosing the best method for removing the unbound dye from extracellular vesicles.

## Graphical abstract

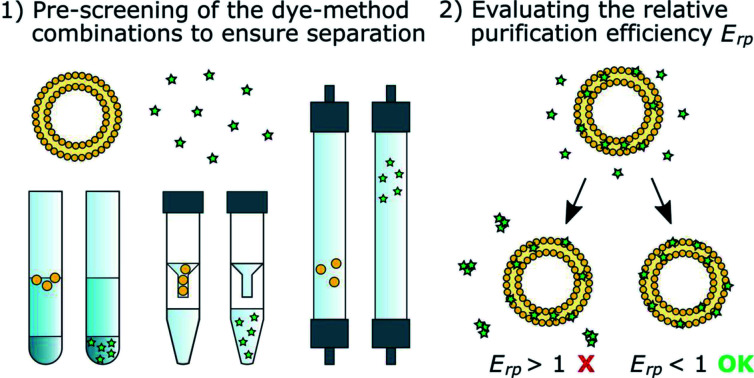

## 1 Introduction

Extracellular vesicles (EVs) have gained significant attention as promising drug carriers for personalized nanomedicine over the recent decade. EVs are structurally heterogenous membrane-bound nanoparticles that are excreted by cells.^1^ EVs have been associated with immune responses,^2^ viral pathogenicity,^3^ central nervous system related diseases,^4,5^ and cancer progression.^6^ Because of the natural origin of the EVs and their unique innate properties as natural cargo, the EV research foresees their potential applications as diagnostic or therapeutic tools for various diseases.

EV trafficking and their interactions with cells, tissues and *in vivo* are typically studied with fluorescence-based microscopy methods.^4,7–9^ Furthermore, fluorescent labelling of EVs can enhance the sensitivity and selectivity of EV characterization methods, *i.e.*, flow cytometry^10,11^ and nanoparticle tracking analysis.^12,13^ There are several different approaches for the EV labelling;^14,15^ the simplest and most commonly used method is the incubation of isolated EVs with lipid-tracer fluorescent dyes, such as long-chain dialkylcarbocyanines, including DiI, DiD^8,16–18^ and PKH dyes.^9,10,17,19^ This is referred to as passive loading of the dyes in contrast to active loading methods^20^ and covalent dye grafting.^21,22^ The passive labelling is a relatively safe alternative among the other labelling methods due to the least effect on the natural EV structure.^23^ Since the covalent labels often react with the amine groups of the proteins at the surface of the EV membrane and active labelling requires extra chemical or physical treatments of EVs, the covalent or active labelling may lead to a reduction of EV unique intrinsic features and a deterioration of their potential as nanocarrier.

Being an equilibrium process, the passive labelling usually results in a mixture of the labelled EVs and an unbound dye in an aqueous solvent.^21,23–26^ Covalent labelling is also often based on equilibrium chemical reactions and thus excess of reactive dye remains in the final labelled sample.^21^ Consequently, in both cases the unbound dye needs to be removed from the labelled EVs. The methods used for this include the same procedures as used for the initial EV isolation, such as ultracentrifugation,^9,19,27^ various filtrations,^8,17^ and size-exclusion chromatography.^16,22^ A successful removal of the unbound dye from the EVs is a crucial step for most EV studies, as the fluorescent dye not associated with the EVs will likely compromise the research outcomes or even lead to wrong conclusions of the EV functionality.^28^

Although the challenges in unbound dye removal have been noticed and discussed earlier, there are no clear criteria for selecting a suitable purification method to remove the excess dye from the labelled EVs, and the success of the purification is rarely estimated. In a few studies, the purification success was confirmed by comparison with free dye controls,^21,24^ while characterization of the labelled EVs was done by quite exotic methods which can hardly be available in all laboratories, *e.g.* asymmetric-flow field-flow fractionation coupled to a multi-angle light-scattering detector,^24^ or nanoscale fluorescence analysis and cytometric sorting.^21^ Although the above methods bring important and convincing information, they can be too laborious for screening of multiple labels, labelling conditions, and suitable purification methods. That is why there is a need for a simpler pre-screening protocol for an assessment of the purification after labelling. In some papers, the EV labelling efficiency was estimated as a relation of the recovered amount of label either to the recovered amount of EVs,^20^ or to the protein content in the labelled EV preparation.^29^ They however lacked an evaluation of the purification efficiency after labelling which clearly determines the accuracy of the labelling efficiency estimation. Conclusively, the lack of a standard way to characterize the purification efficiency makes published work difficult to compare. Therefore, there is a clear need for a systematic comparison of the purification methods.^15^ The present work is our attempt to fulfil this urgent need.

**Scheme 1:**
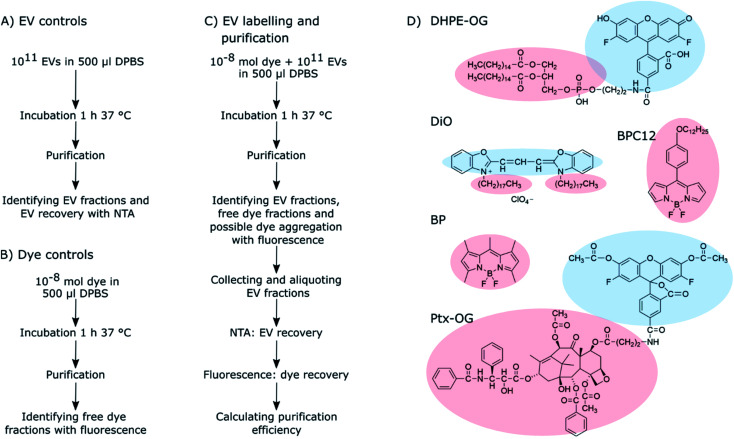
The workflow of the (A) EV control, (B) dye control, and (C) EV labelling and purification experiments. The details of these experiments are described in Sections 2.3–2.6. (D) The molecular structures of the fluorescent dyes used for the EV labelling. Light red clouds point out the hydrophobic parts of the molecules intercalating into the EV lipid membrane, and light blue ones indicate more hydrophilic parts probably located in the outer surface of the EVs exposed to the surroundings.

In this study, we focused only on purification method selection and estimation of purification efficiency and did not study EV labelling efficiency. Five widely available purification methods were studied for their ability to separate fluorescently labelled EVs from unbound dye: ultracentrifugation (UC), ultracentrifugation with discontinuous density gradient (UCG), ultrafiltration (UF), size exclusion chromatography (SEC) and anion exchange chromatography (AEC). Five fluorescent dyes were used for the passive EV labelling, resulting in 25 dye – purification method combinations. The overall workflow of the study and the molecular structures of the studied dyes are presented in Scheme 1. First, the behaviour of the dyes and the EVs were studied separately with each of the methods to screen the potential methods offering sufficient separation between the dyes and the EVs. Based on these control results, potential purification methods were chosen for each dye, and they were subsequently applied to the labelled EVs. The relative purification efficiency was estimated based on the EV and dye recovery for each sample, and the results were compared for different purification methods and dyes. Finally, the labelled and purified EVs were applied to cells and imaged by fluorescence lifetime microscopy.

As clearly seen from the chemical structures of the dyes, they are all significantly hydrophobic and, thus, should intercalate into the EV membrane in aqueous solution (Scheme 1D). DHPE-OG is a fluorescent conjugate of a lipid molecule. Being able to label EVs with lipids would be beneficial since they are natural components of the EV membrane and thus useful *e.g.*, for adding targeting units to EVs.^30^ DiO was chosen since carbocyanine dyes are commonly used in membrane staining.^31^ BPC12 is a molecular rotor dye and its viscosity dependent fluorescence could be used to study the integrity of the EVs during cell up take and consequent trafficking inside the cell.^32^ BP is a neutral, nonpolar lipid stain.^33^ Both these BP dyes are very hydrophobic and will thus intercalate deep into the EV membrane.^34^ All the other studied dyes are at least partially at the hydrophilic part of the EV membrane and thus exposed to the surroundings. Hence, they can influence the EV functionality and especially the cell uptake.^35^ As an example of loading EVs with a biologically active molecule, the labelling was also studied with a Ptx-OG, a tubulin targeting anti-cancer agent Paclitaxel labelled with fluorescent dye OG.^7,36^ Being somewhat hydrophobic, Ptx part will intercalate into the EV membrane.

## 2 Materials and methods

### 2.1 EV isolation

PC-3 cell line was obtained from the American Type Culture Collection (ATCC, USA) and cultured in the CELLine AD 1000 bioreactor (Sigma-Aldrich, USA) at 37 °C and 5% of CO_2_. The cell culture compartment was filled with 15 ml Advanced DMEM/F-12 glucose (4.5 g ml^−1^) and l-glutamine (2 mM), while the media compartment was filled with 750 ml Ham's F-12k medium supplemented with 10% fetal bovine serum (FBS) and glucose (4.5 g ml^−1^). Cell culture compartment and media compartment are devised by a semi-permeable membrane (molecular weight cut-off 10 kDa), which permits a continuous nutrient diffusion and waste elimination. The membrane prevents the diffusion of EVs and large proteins from one compartment to the other. Cell culture media, FBS and l-glutamine were purchased from ThermoFisher Scientific (USA) and the glucose from Sigma-Aldrich (USA).

PC-3 cell derived extracellular vesicles were isolated from the cell culture media by differential ultracentrifugation. First, buoyant cells, cell fragments and apoptotic bodies were removed with low-speed centrifugation at 2500×*g* for 25 min at +4 °C (Eppendorf Centrifuge, rotor FA-45-6-30, Germany). Next, the first EV fraction (20k EVs) was pelleted with centrifugation at +4 °C with 20 000×*g* (12 741 rpm, *k*-factor 1287) for 1 h (38.5 ml, Open-Top Thinwall Polypropylene Tube, Optima L-80 XP ultracentrifuge with SW 32 Ti Rotor, Beckman Coulter, USA). A second EV fraction (110k EVs) was collected from the supernatant with centrifugation at +4 °C with 110 000×*g* for 2 h (29 881 rpm, *k*-factor 234).

Next, the EV pellets were resuspended in DPBS (Dulbecco's phosphate buffered saline, Sigma-Aldrich, USA) buffer and the EVs were further purified with three-layered (0% to 35% to 45%) discontinuous iodixanol density gradient. The EV suspension was mixed with iodixanol (Optiprep™, Alere Technologies AS, Norway) to a final volume of 2 ml and a 45% iodixanol concentration and loaded to the bottom of the density gradient. 4 ml of 35% iodixanol was placed over the bottom layer, and the rest of the tube was filled with DPBS. The gradient was centrifuged at +4 °C with 200 000×*g* (40 291 rpm, *k*-factor 129) for 3.5 h (13.2 ml, Open-Top Thinwall Ultra-Clear Tube, Optima L-80 XP ultracentrifuge with SW 41 Ti Rotor, Beckman Coulter, USA). Upon the centrifugation the EVs move from their original bottom layer (density of >1.215 g ml^−1^) to the top of the layer with the density of ∼1.195 g ml^−1^ with a velocity dependent on the cube of their size,^37^*i.e.*, the EVs concentrate on the 0–35% iodixanol interface according to their buoyant density. DPBS on top of the gradient was discarded, and the EVs were collected from the 0–35% iodixanol interface. Finally, the iodixanol was removed by serial ultrafiltration (Amicon Ultra-15 Centrifugal Filter units, cut-off 10 kDa, Millipore, USA) at 5000×*g*, 20 min, +4 °C (Eppendorf Centrifuge, 5810 R fixed-angle rotor Hamburg, Germany). The EV suspension was divided to aliquots of 10^11^ particles and stored at −80 °C until they were used for the experiments. In this study, the EV populations are classified as the 20k EVs and the 110k EVs, according to the centrifugal forces used for their isolation.

### 2.2 Characterization of the isolated EVs

The isolated EVs were characterized according to MISEV2018 standards^38^ by western blot, transmission electron microscopy and ATR-FTIR as described in (ESI Section S1†). The particle concentrations and the size distributions of the isolated EV fractions were analysed using the NanoSight LM-14 instrument (LCM14C, 405 nm laser, 60 mW, Nanosight, Salisbury, United Kingdom), equipped with the sCMOS camera (Hamamatsu Photonics K.K., Hamamatsu, Japan). The samples were diluted with DPBS and measured using a camera level 15 and an acquisition time of 90 s. Every sample was measured in triplicates. The resulting videos were analysed using the NanoSight NTA software (NanoSight Ltd., v. 3.0) with a detection threshold set to 5. Noteworthy, the NTA instrument can detect only particles larger than 80 nm. After the NTA analysis, the EV samples were divided to aliquots of 10^11^ particles and stored frozen in −80 °C until they were used for labelling. Similar NTA analysis was done for all the labelled EVs and non-labelled EV controls after every purification for estimating EV recoveries (Scheme 1A and C).

### 2.3 Passive labelling of EVs

Five different fluorescent dyes were used for the EV labelling: Oregon Green™ 488 1,2-dihexadecanoyl-*sn*-glycero-3-phosphoethanolamine (DHPE-OG), 3,3′-dioctadecyloxacarbocyanine perchlorate (DiO), 4,4-difluoro-1,3,5,7,8-pentamethyl-4-bora-3*a*,4*a*-diaza-*s*-indacene (BP), 4,4′-difluoro-4-bora-3*a*,4*a*-diaza-*s*-indacene *meso*-substituted with *para*-dodecylphenyl moiety (BPC12),^32^ and a tubulin tracer dye Oregon Green 488 taxol, bis-acetate (trade name Tubulin Tracker Green; here, Ptx-OG). BPC12 was synthetized in our group as described in the literature,^32^ and the other dyes were purchased from ThermoFisher Scientific (USA). The dye stocks were prepared in dimethyl sulfoxide (DMSO, Sigma-Aldrich, Germany). The emission properties of the dyes were studied without EVs in two different solutions: DPBS and solubilized in DPBS by addition of 1% Triton X-100 (Surfact-Amps X-100, ThermoFisher Scientific, USA) with spectrofluorometer (FluoroLog-3, Horiba Scientific, Japan; excitation wavelength 483 nm, emission range 500–800 nm) to find possible aggregation-related emission bands. For the dyes showing clear aggregate emission, the emission spectra were measured also for the purified EVs as one criteria of the purification quality.

To be able to study the purification abilities of the chosen methods, the dye-to-EV ratio was chosen to have a significant excess of the fluorescent dye. EV aliquots of 10^11^ particles were diluted to a final volume of 0.5 ml in DPBS, and 10^−8^ mol of dye was added to the EVs while mixing with a vortex mixer to prevent immediate aggregation of the hydrophobic dye in aqueous buffer solution. The final DMSO concentration in the EV suspension was less than 2.5%. The labelling mixture was then incubated for 1 h at 37 °C upon shaking while protected from light (Scheme 1C). All the EV labelling was done in triplicates.

### 2.4 Purification methods

Five widely available purification methods were studied for their ability to separate the labelled EVs from the unbound dye: ultracentrifugation (UC), ultracentrifugation with discontinuous iodixanol density gradient (UCG), ultrafiltration (UF), size exclusion chromatography (SEC), and anion exchange chromatography (AEC). In a successful UC purification, the EVs pellet during the centrifugation while the unbound dye stays mostly in the supernatant and can be carefully aspirated above the EV pellet. In UCG, because of their buoyant density and bigger size compared to fluorescent dye molecules, the EVs float to the higher interface between the layers with smaller densities while the unbound dye is expected to stay mostly at the bottom of the gradient in the highest density layer. Both UF and SEC are based on the size separation: in UF, the EVs concentrate above the filter membrane as most of the unbound dye is washed to the filtrate, and in SEC, the EVs elute from the column before the unbound dye. In AEC, the EVs first bind to the positively charged column and the unbound dye is washed from the column; then, the EVs are released from the column with buffer containing NaCl.

#### 2.4.1 Ultracentrifugation

The labelled EV suspension was diluted to a 1 ml final volume with DPBS. Two different centrifugation parameters were used in accordance with the parameters used for the initial EV isolation: the 20k EVs were pelleted with centrifugation at +4 °C with 20 000×*g* (18 185 rpm, *k*-factor 289) for 1 h, and the 110k EVs were pelleted with centrifugation at +4 °C with 110 000×*g* (42 647 rpm, *k*-factor 53.0) for 2 h. The centrifugations were done by an Optima MAX ultracentrifuge equipped with the MLA-130 fixed angle rotor in 1 ml Open-Top Thickwall polycarbonate tubes (Beckman Coulter, USA). The supernatant was aspirated just above the formed pellet to avoid disturbing the rather loose pellet, and the EVs were allowed to disperse in 70 μl of DPBS overnight at +4 °C.

#### 2.4.2 Ultracentrifugation with density gradient

The UCG purification was done similarly as in the EV isolation step. The centrifugation was done at +4 °C with 200 000×*g* (40 291 rpm, *k*-factor 129.0) for 3.5 h by an Optima XPN ultracentrifuge, equipped with a SW 41 Ti swinging bucket rotor (Beckman Coulter, USA). After collecting 1 ml fractions (a total of 13–14 fractions), the fluorescence of the collected fractions was measured for identifying the EV and the free dye fractions. The fluorescence experiments were done either by a spectrofluorometer (FluoroLog-3, Horiba Scientific, Japan) or by a fluorescence plate reader (Fluorescent Ascent FL, Thermo Scientific, USA). Based on the NTA results at EV isolation step (Section 2.1) and in the EV controls (Section 2.5), the highest particle concentrations were expected to be in the interface between DPBS and 35% iodixanol. Fluorescence intensity registered in 1–2 fractions on this interface verified their choice as the EV fractions. These fractions were either pooled and the iodixanol was removed by serial ultrafiltration as described in Section 2.4.3 (UCG + UF), or the pooled EV fractions were analysed without removing the iodixanol (UCG).

#### 2.4.3 Ultrafiltration

The labelled EV suspension was diluted to a final volume of 4.5 ml with cold DPBS and concentrated with a 10 kDa membrane (Microsep Advance Centrifugal Device, Omega membrane) by centrifugation with Sigma 4-16KS centrifuge (Sigma Laborzentrifugen GmbH, Germany) at +4 °C. The centrifuge parameters were adjusted to yield a maximum final volume of 0.5 ml after each washing round. The filtrate was collected, and the washing was repeated a total of 3–6 times. After the serial ultrafiltration, the EV-containing sample was carefully collected above the membrane by gentle pipetting.

#### 2.4.4 Size-exclusion chromatography

The labelled EV suspension was run through a Sepharose CL-2B (Cytiva, USA) column (diameter 1 cm, bed size ∼13 ml) using DPBS as an eluent. All the runs were performed at room temperature. Starting directly after the sample insertion, eluted buffer was collected in 1 ml fractions. A total of 30 fractions were collected for each SEC run. The EVs eluted typically in fractions 4 and 5 (Section 2.5). After every SEC run with the fluorescent dyes, the dye retained in the column was washed with 25 ml of 0.1% Triton X-100 (Surfact-Amps X-100, ThermoFisher Scientific, USA) in 0.1 M NaOH, followed by extensive washing with Milli-Q water. The fluorescence of the collected fractions was measured with a spectrofluorometer for determining the fluorescent dye distribution and identifying the fractions containing the labelled EVs for further analysis.

#### 2.4.5 Anion exchange chromatography

**Fig. 1:**
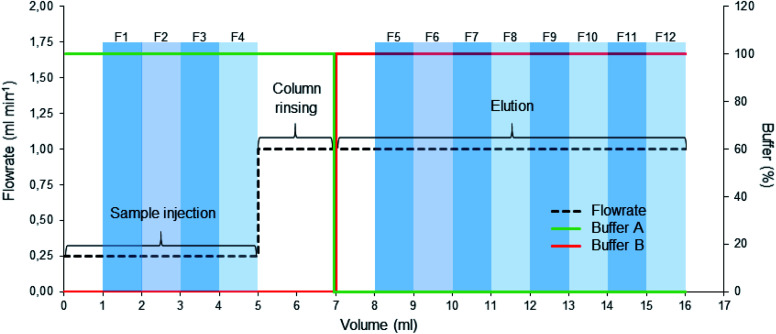
AEC running protocol. After preparing the column, the sample was injected at 0.25 ml min^−1^ (0–5 ml) and washed with the start buffer (100% A), collecting fractions (1–4) after one column volume (1 ml). The elution was then started with buffer B, collecting fractions (5–12) after one column volume had passed, followed by washing and regeneration of the column.

A 1 ml HiTrap® DEAE Fast Flow anion exchange column (Sigma-Aldrich, Germany) was used. The column was connected to the NGC Quest Plus chromatography system (Bio-Rad, USA) equipped with a fraction collector (kept at +4 °C) and a 1 ml sample injection loop. Two running buffers (A and B) at pH 7.5 were prepared. Buffer A was composed of 30 mM Tris–HCl (Sigma-Aldrich) and buffer B of 30 mM Tris–HCl and 1 M NaCl (Sigma-Aldrich). All the runs were performed at room temperature with the running protocol presented in Fig. 1. To reduce the non-specific binding of EVs to the column, the column was treated with 2.5% (w/v) BSA solution (Sigma-Aldrich) in buffer B before each run. 3 ml of BSA in buffer B was injected into the column and incubated for 30 minutes, followed by washing with 15 ml of buffer B at 1 ml min^−1^ and 8 ml of buffer A at 1 ml min^−1^. 0.5 ml fractions were collected during the injection (4 fractions, 1–4) and elution (8 fractions, 5–12) phases to analyse the flow-through as well as the eluent (Fig. 1). After each run, the column was washed by injecting 3 ml of 0.1% Triton X-100 – 1 M NaCl followed by 10 ml of buffer B at 1 ml min^−1^. The EVs eluted typically in fractions 6–12, with peak concentration in fraction 7 (Section 2.5) as identified with NTA analysis. Similarly to SEC, the fluorescence of the collected fractions was measured with a spectrofluorometer for determining the dye distribution.

### 2.5 Control purifications

Before using any of the purification methods for the EVs incubated with a fluorescent dye, the behaviour of the dye was studied without the EVs (Scheme 1A). Conversely, the EV behaviour and recoveries in all the methods were studied also without the fluorescent dyes (Scheme 1B). The dye and EV controls were used to select the methods with potential to separate the unbound dye from the EVs. Dye controls were prepared by diluting 10^−8^ mol of the dye to a final volume of 0.5 ml in DPBS; similarly, the non-labelled EV controls were prepared by diluting EV aliquots of 10^11^ particles to a final volume of 0.5 ml of DPBS (Scheme 1). Both control samples were then incubated and purified in the same way as the labelled EV suspensions. The EV controls were performed in triplicates and the dye controls once.

In the EV controls, the expected EV fractions were collected and analysed with NTA for determining the EV recoveries (Section 2.6). In the chromatography methods, the EV containing fractions were identified by analysing fractions 1–10 (SEC) or 1–12 (AEC) with NTA for the first of the EV control replicates. Based on the results for the rest of the replicates only the EV containing fractions were analysed. With UCG, the majority of the unlabelled EVs were in the 0–35% iodixanol interface. In higher-density fractions, the scattering background from iodixanol made the NTA analysis unreliable, and therefore those fractions were not analysed with NTA.

For the dye controls, fluorescence was used for studying whether the dyes behave as expected for a successful purification. For the UC and UF dye controls, the dye recovery *R*_dye,c_ was estimated by comparing fluorescence in the expected EV fraction (resuspended UC pellet and UF retentate) to the total fluorescence of the sample, measured with the spectrofluorometer:1
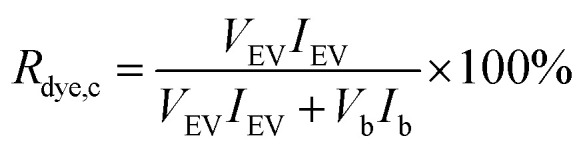
where *V*_EV_ is the volume and *I*_EV_ the fluorescence intensity of the expected EV fraction, and *V*_b_ and *I*_b_ correspondingly the volume and fluorescence intensity of the buffer that is not expected to contain the EVs (UC supernatant and UF filtrate). For the fraction-based methods (UCG, SEC and AEC), the potential separation was determined by identifying dye-containing fractions by fluorescence experiments and comparing these to the corresponding EV controls. The fluorescence was measured either with a fluorescence plate reader (UCG controls for BPC12, DHPE-OG and Ptx-OG) or with a spectrofluorometer (all the remaining controls).

### 2.6 Characterization after purification

After the purification, all the EV samples were divided into aliquots and stored at −80 °C for the characterization (Scheme 1C). The particle concentrations of the EV suspensions recovered after labelling and purification were measured with NTA to obtain EV recovery (*R*_EV_):2
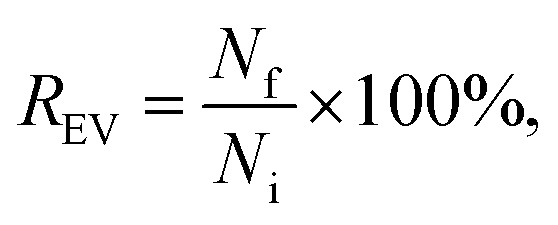
where *N*_f_ is the final number of particles recovered after the purification and *N*_i_ = 10^11^ is the number of particles initially used for EV labelling before the purification process.

The dye concentration in the purified samples was measured against a dye calibration curve either with a plate reader (Ptx-OG, DHPE-OG, BPC12, DiO, BP UCG and BP AEC) or with a fluorescence spectrophotometer (BP UC and BP UF). The fluorescence of the samples with known amount of a dye were measured to form the calibration curve. The dyes were released from the EVs and solubilized in the buffer by adding 1% Triton X-100 to both the EV and the calibration samples. Ptx-OG was hydrolysed to its fully fluorescent form by adding sodium hydroxide (0.015 M final concentration) together with 1% Triton X-100 to both the EV and the calibration samples and incubating at 37 °C for 1 h before the measurements. Dye recovery *R*_dye_ was calculated as3
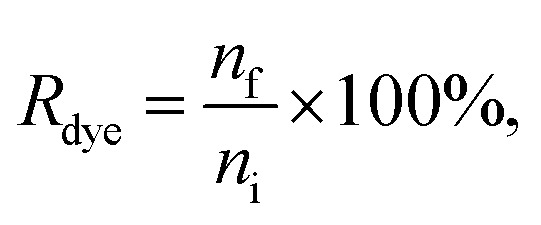
where *n*_f_ is the molar amount of dye left in the recovered EV-containing sample, and *n*_i_ is the molar amount of the dye initially added to the EV suspension for the labelling.

To compare the purification result between the different methods and the different dyes, relative purification efficiency *E*_rp_ was calculated as4
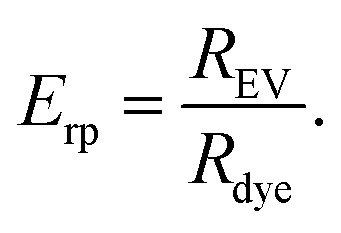


Given that a significant excess of dyes was always used, *E*_rp_ < 1 indicates that the method concentrates the unbound dye more efficiently than the EVs and therefore it is not suitable for the purification of the EVs after fluorescent labelling. For the methods yielding *E*_rp_ > 1, the relative purification efficiency was used for comparing the suitability of the purification methods for each dye.

### 2.7 FLIM imaging of cells incubated with EVs

One day before imaging, 10 000 PC-3 cells were seeded in 70 μl 2-well inserts (Ibidi, Germany) attached to a glass-bottom 35 mm Petri dish (poly-d-lysine coated, no. 1.5 coverslip, 10 mm glass diameter, MatTek, USA), or 75 000 cells were seeded directly on the Petri dish. Cells were incubated with labelled and purified EV sample (30 000–400 000 particles/seeded cell) for 3 hours, washed once with DPBS and imaged in FluoroBrite DMEM (Gibco, USA) supplemented with 10% (v/v) FBS. Samples for the free dye control were prepared similarly, using free dye instead of the labelled EVs. Similar amount of free dye was added to the cells as would have been added with 30 000 particle/cell – ratio.

Fluorescence lifetime images were acquired using the fluorescence lifetime microscope MicroTime-200 (PicoQuant, Germany) coupled to the inverted microscope Olympus IX-71 (Olympus, Japan) equipped with 100× oil objective (NA = 1.4). The pulsed laser diode LDH-P-C483 (PicoQuant, Germany) emitting at 483 nm (time resolution 120 ps) was used for the excitation and the emission was monitored on wavelengths 510–900 nm. The samples were imaged at 37 °C and 5% CO_2_ using an objective heater (TC-1-1005 Temperature Controller, Bioscience Tools, USA) and a custom-made incubator. The FLIM images were analysed in SymPhoTime 64 software (PicoQuant, Germany). The colours of the FLIM images are based on the mean arrival times of the emitted photons after the excitation pulse (fast lifetime). The intensity-averaged lifetimes *τ*_av_ were obtained by 2- or 3-exponential lifetime fitting^7^ (DHPE-OG and DiO, respectively) of the decay curves of the selected regions of interest, excluding the cell autofluorescence background.

## 3 Results

### 3.1 EV characterization

Two different subpopulations of PC-3 EVs, 20k and 110k, were isolated from conditioned media by differential ultracentrifugation, followed by density gradient centrifugation. The isolated EVs were characterized by NTA, TEM, FTIR and WB analysis. The detailed characterization is presented in ESI Section S1.† Briefly, the isolated EVs were confirmed to be of high purity having consistently similar properties over each sample replicate regarding size, enrichment of the EV-associated proteins and overall biochemical composition according to FTIR spectroscopy. The most apparent differences between 20k and 110k EVs were that 20k EVs were larger on average, which is in line with their consequently faster sedimentation during 20 000×*g* centrifugation, and the more intense peaks in the 1040–1110 cm^−1^ region in the FTIR spectra for the 110k EVs.

### 3.2 Control purifications

#### 3.2.1 Recoveries of non-labelled EVs

First, the EV recoveries after each purification method were measured without fluorescent dyes (Scheme 1A) by NTA (Table 1). These non-labelled EV controls were prepared and incubated similarly as the fluorescently labelled EVs. Regardless of the purification method, a significant number of the EVs was lost in the purification process and the *R*_EV_ between replicates had high variation. The highest *R*_EV_ values were obtained by UCG and AEC: both methods were able to recover over 45% of the EVs. Without BSA blocking of the column, the AEC yields were also low (approximately 10%, data not shown). This indicates that the non-specific binding of the EVs to the column was significantly reduced by BSA blocking. In UC and SEC, the *R*_EV_ is moderate, from 10% to less than 35% in average. In contrast, the *R*_EV_ was the lowest when the gradient centrifugation was followed by the ultrafiltration step (UCG + UF). This indicates high EV binding to the filter membrane, which is also reflected in the *R*_EV_ after direct ultrafiltration (UF). Because of the very low EV recoveries in UCG + UF (about 1%), the UCG purification of labelled EVs was studied only without removing the iodixanol. Interestingly, an absorption peak characteristic of iodixanol was observed in the AEC during the sample injection (1–5 ml, data not shown), indicating effective removal of residual iodixanol from the EVs.

**Table 1:** Recoveries *R*_EV_ of non-labelled EVs after different purification methods: ultracentrifugation (UC), ultracentrifugation with density gradient without ultrafiltration (UCG) and with ultrafiltration (UCG + UF), ultrafiltration (UF), size-exclusion chromatography (SEC), and anion exchange chromatography (AEC). The individual values for each replicate are presented in ESI Table S1

Method	110k, *R*_EV_ (%)	20k, *R*_EV_ (%)
UC	12.2 ± 3.0	24.0 ± 11.0
UCG	82.3 ± 11.6	59.5 ± 6.2
UCG + UF	0.6 ± 0.1	1.3 ± 0.3
UF	4.3 ± 3.2	11.1 ± 15.2
SEC	33.8 ± 16.3	10.2 ± 5.9
AEC	64.7 ± 6.9	45.9 ± 9.0

#### 3.2.2 Dye distributions in control purifications without EVs

**Fig. 2:**
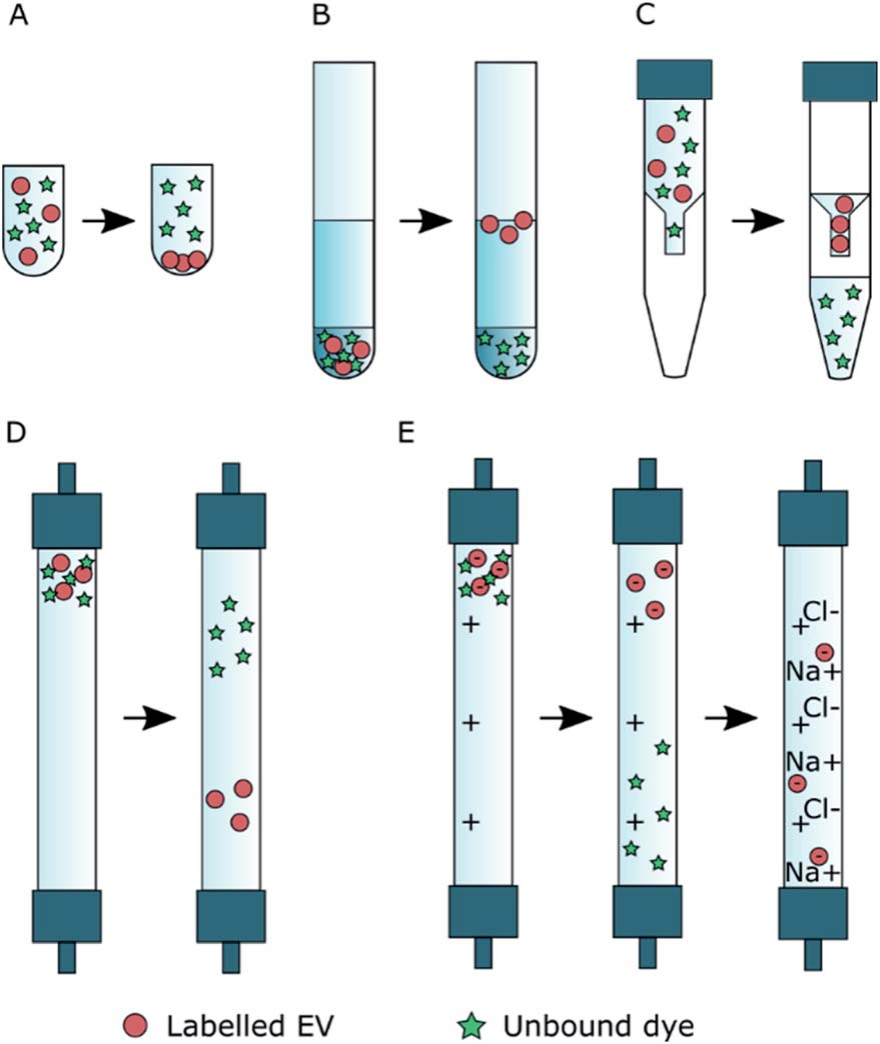
The expected separation principles for a successful EV separation from non-EV-bound dye. (A) Ultracentrifugation: the EVs pellet during the centrifugation while the unbound dye stays in the supernatant; (B) ultracentrifugation with density gradient: the EVs float to the higher interface between the layers with smaller densities, and the unbound dye stays at the bottom of the gradient in the highest density layer; (C) ultrafiltration: the EVs concentrate above the filter membrane as the unbound dye is washed to the filtrate; (D) size exclusion chromatography: the EVs elute from the column before the unbound dye; (E) anion exchange chromatography: first, the EVs are bound to the positively charged column and the unbound dye is washed from the column; then, the EVs are released from the column with buffer containing NaCl.

For understanding the purification potential of a particular method, it is important to know what happens to the unbound dye in the purification process. Thus, the behaviour of each dye in the absence of EVs was evaluated for all the purification methods (Scheme 1B). The expected behaviour is schematically presented in Fig. 2.

##### 3.2.2.1 Ultracentrifugation and ultrafiltration (Fig. 2A and C)

For the UC and UF dye controls, the dye recoveries in the expected EV fractions (UC pellet and UF retentate) were estimated from the fluorescence intensity of resuspended pellet and supernatant (UC, Fig. 2A), or from the sample collected above the filter membrane and the highest concentration filtrate (UF, Fig. 2C) according to eqn (1). Thus, the optimal *R*_dye,c_-value would be ∼0 for the dye controls. The obtained *R*_dye,c_-values are presented in Table 2.

**Table 2:** Ultracentrifugation (UC) with two centrifugation forces (110 000×*g* and 20 000×*g*) and ultrafiltration (UF) dye control results. The dye recoveries in the expected EV fractions *R*_dye,c_ were estimated from fluorescence spectra measured at 550 nm (DiO), 515 nm (BP) and 522 nm (DHPE-OG and Ptx-OG)

Dye	UC 110k, *R*_dye,c_ (%)	UC 20k, *R*_dye,c_ (%)	UF, *R*_dye,c_ (%)
DHPE-OG	3	4	9
Ptx-OG	52	59	96
BP	<1	1	2
BPC12	—	—	—
DiO	80	65	97

DiO and Ptx-OG had virtually no fluorescence in the UC supernatant and UF filtrates, while the emission intensity in the resuspended UC pellet and the UF retentate was high. The result clearly shows that these dyes aggregate and, therefore, they are efficiently pelleted during UC and do not pass the filter membrane in UF. Consequently, these methods are not suitable for removing DiO or Ptx-OG from the labelled EVs.

BP and DHPE-OG gave better control results. DHPE-OG fluorescence was still clearly detected both in the UC pellet and the UF retentate (*R*_dye,c_ = 3–9%), while almost no BP fluorescence was observed (*R*_dye,c_ = 1–2%). Because of the low dye recoveries, BP was chosen as a model dye for the UC and UF purifications.

BPC12 stuck to the centrifuge tube walls during the ultracentrifugation and attached to the UF filter device membrane showing orange colouring on the tube walls and the filter membrane. The detected fluorescence intensities were low even upon addition of surfactant (1% Triton X) to solubilize the dye, indicating strong surface adsorption. Since UC and UF did not work as expected with BPC12, the dye recoveries could not be reliably estimated. However, the possibility of using these methods to purify EVs from unbound BPC12 based on the dye adsorption on the surfaces was studied further with BPC12-labelled EVs.

##### 3.2.2.2 Ultracentrifugation with density gradient (Fig. 2B)

**Fig. 3:**
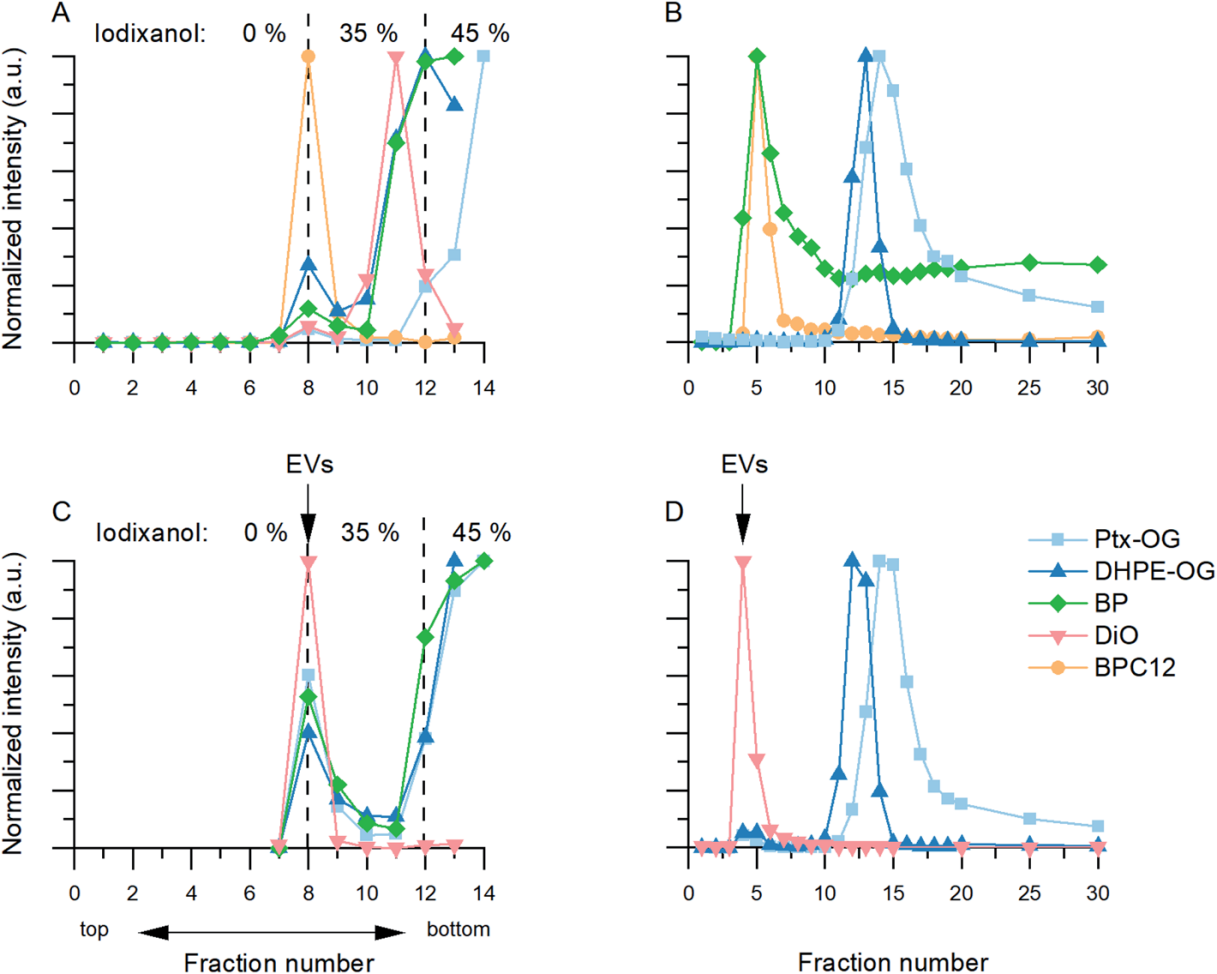
Dye control results and examples of EV fluorescence distributions for the UCG (A and C) and SEC purification (B and D). In all graphs, normalized fluorescence intensity is presented as a function of fraction number. The same colour scale has been used in all graphs. (A) UCG control. The iodixanol concentration is presented in the top of the figure. (B) SEC control. DiO did not pass the SEC column and is therefore not shown here. (C) Fluorescent dye distribution in UCG fractions with labelled 110k EVs. (D) Fluorescent dye distribution in SEC fractions with labelled 110k EVs.

In the UCG controls, most of the Ptx-OG, DHPE-OG and BP stayed in the bottom fractions (45% iodixanol) of the gradient (Fig. 3A). During the centrifugation, DiO formed visible crystals that did not concentrate in 45% iodixanol layer but had migrated further in the 35% iodixanol layer (Fig. 3A, pink). With all these dyes, there was also some emission at the interface between 0% and 35% iodixanol, where also the EVs concentrate according to the unlabelled EV controls. However, as most of the free dye stayed in higher density fractions and the EV recoveries before UF were high (Table 1), UCG purification was studied further for the EVs labelled with Ptx-OG, DHPE-OG, BP and DiO. In contrast, BPC12 emission was observed only in the fractions where EV accumulation is expected (Fig. 3A, yellow), implying that UCG is not suitable method to purify the BPC12 labelled EVs due to similarities in the dye and EV particle density.

##### 3.2.2.3 Size-exclusion chromatography (Fig. 2D)

Three types of dye behaviour were observed in the SEC controls. Both BODIPY dyes eluted in the same fractions as EVs (Fig. 3B, green and yellow). Most of the BPC12 eluted in fractions 4 and 5, indicating that it forms particles large enough for size exclusion to occur in the column. Separate NTA controls confirmed that BPC12 forms particles with about 90 nm diameter in DPBS (ESI Fig. S4†). Although BP did not form particles visible by NTA (ESI Fig. S4†), the highest concentrations of BP eluted in the fractions 4–7, indicating the formation of particles too small for NTA to detect, but large enough for the size exclusion effect, followed by a high background level in later fractions. Instead, both Oregon Green dyes had good separation from EVs and started eluting first after fraction 10 (Fig. 3B, blue and light blue). DiO, being the least water soluble of the dyes in this study, did not pass the column at all: instead, it crystallized and stayed on top of the column. Based on the control results, SEC purification was studied further with Ptx-OG, DHPE-OG and DiO.

##### 3.2.2.4 Anion exchange chromatography (Fig. 2E)

Since both OG dyes (Ptx-OG after hydrolysation) contain a negatively charged carboxylic acid group, their separation with AEC was expected to be challenging. Neutral dyes (BODIPY dyes) and positively charged dyes (DiO) were expected to elute in the sample injection step. Unfortunately, the AEC principle turned out to be unsuitable for the studied dyes. Based on the dye controls, all of the OG and BP dyes elute in the same fractions as the EVs (ESI Fig. S5†). DiO had the most promising result, giving only negligible fluorescence in the expected EV fractions. Although it was not removed in the sample injection step as expected, DiO crystallized similar to the SEC DiO control and did not pass the column at all. Consequently, IEC was studied only for the purification of the DiO-labelled EVs.

### 3.3 EV purification results

The chosen purification methods for each dye were applied to the labelled EVs. The EVs were incubated with a constant concentration of fluorescent dye in DPBS as described in Section 2.3 and Scheme 1C. The EV and dye recoveries (eqn (2) and (3)) as well as the relative purification efficiencies (eqn (4)) were used to estimate the quality of the purification (Table 3). In general, labelling decreased the EV recoveries (Table 3) compared to the unlabelled controls (Table 1). DiO- and Ptx-OG-labelling reduced the recoveries the most with all the used purification methods. On the other hand, BP-labelled EVs had almost the same *R*_EV_ after UCG as the unlabelled EVs.

**Table 3:** EV recoveries *R*_EV_, dye recoveries in the EV fractions *R*_dye_, and relative purification efficiencies *E*_rp_ for the labelled and purified EVs. The removal of unbound dye was studied with ultracentrifugation (UC), ultracentrifugation with density gradient without ultrafiltration (UCG), ultrafiltration (UF), size-exclusion chromatography (SEC), and anion exchange chromatography (AEC). The individual values for each replicate are presented in ESI Table S2

Dye	Method	110k EVs			20k EVs		
		*R*_EV_ a (%)	*R*_dye_ (%)	*E*_rp_ a ^,^ b	*R*_EV_ a (%)	*R*_dye_ (%)	*E*_rp_ a ^,^ b
DHPE-OG	UCG	43.0 ± 2.8	44.6 ± 4.2	1.0	52.9 ± 7.5	39.6 ± 3.3	1.3
	SEC	12.2 ± 1.6	8.7 ± 1.4	1.4	8.2 ± 0.9	9.3 ± 5.0	0.9
Ptx-OG	UCG	10.3 ± 0.4	67.8 ± 11.5	0.2	6.5 ± 3.3	41.3 ± 38.4	0.2
	SEC	3.8 ± 1.6	2.9 ± 1.3	1.3	3.7 ± 0.9	1.3 ± 0.3	2.8
BP	UC	7.6 ± 4.8	16.6 ± 1.3	0.5	<1	7.0 ± 0.8	—
	UF	1.2 ± 0.7	1.8 ± 0.9	0.7	2.3 ± 1.0	4.8 ± 3.8	0.5
	UCG	78.6 ± 10.3	6.2 ± 0.9	12.7	54.0 ± 6.0	15.5 ± 4.8	3.5
BPC12	UC	12.5 ± 6.8	35.5 ± 37.6	0.4	5.9 ± 6.2	17.2 ± 15.3	0.3
	UF	3.9 ± 2.8	7.9 ± 3.4	0.5	8.4 ± 11.2	9,5 ± 15,4	0.9
DiO	UCG	n.d.	n.d.	—	n.d.	n.d.	—
	SEC	1.1 ± 0.2	n.d.	—	<1	n.d.	—
	AEC	10.1 ± 12.3	2.2 ± 2.0	4.6	6.4 ± 0.3	1.8 ± 0.3	3.5

a Colour code: green – acceptably high; red – unacceptably low; black – acceptable with caution.

b *E*_rp_ > 1 indicates successful separation of the labelled EVs from the unbound dye: the greater *E*_rp_, the better separation; conversely, *E*_rp_ < 1 indicates unsuccessful removal of the dye.

#### 3.3.1 Relative purification efficiency

For the comparison of the purification results between different methods, both *R*_EV_ and *R*_dye_ were considered. For an optimal result, *R*_EV_ should be as high as possible (close to 100%). The optimal value of *R*_dye_, however, is more difficult to estimate, as it depends on the labelling efficiency (how many dye molecules are actually located in the EV) and also on *R*_EV_. Consequently, the relative purification efficiency considering both recoveries simultaneously was used for comparing the purification results.

The dyes in this study were relatively hydrophobic, and thus they were expected to locate mainly in the EV membrane after successful labelling. About 60 000 dye molecules were added per each EV in the initial labelling suspension. By estimating the EV membrane area and how many lipid molecules it can accumulate,^39,40^ the dye-to-lipid molar ratio in the labelled EV suspension before purification was at least 1 : 3. As an example, for paclitaxel-loaded liposomes, drug-to-lipid ratios of 1 : 20–1 : 33 have been reported.^41,42^ Such high dye-to-lipid ratios are unlikely reached by passive labelling, and it can be considered that a large excess of dye related to the EVs was used in the present experiments. Therefore, a successful purification process would remove most of the initial dye and a minimum requirement for the purification is that *R*_dye_ < *R*_EV_, yielding *E*_rp_ > 1. In other cases, the dye-to-EV ratio would actually increase during the purification.

#### 3.3.2 DHPE-OG, Ptx-OG, and BP-labelled EVs

For both Oregon Green dyes, UCG and SEC gave the most promising dye control results, and therefore they were used for purifying the labelled EVs. For DHPE-OG EVs, *E*_rp_ shows variation in the purification result: on average, UCG provided better purification for the 20k EVs and SEC for the 110k EVs. In the SEC purification of 20k EVs, *E*_rp_ < 1 for the averaged values although two of the three samples gave *E*_rp_ > 1.3 (ESI Table S2†), which is well in line with the UCG result. This clearly shows the importance of checking the purification quality separately for each sample.

Although the purification efficiencies were similar for both methods, the EV yields were higher after UCG (>40%) than SEC purification (about 10%).

In contrast to DHPE-OG, UCG did not separate the unbound dye from the Ptx-OG-labelled EVs. Only 10% or less of the EVs were recovered, while these fractions contained 40–70% of the fluorescent dye. Instead, the SEC purification gave promising and even reproducible results: although *R*_EV_ was less than 4% for both EV types, *E*_rp_ was 1.3 (110k EVs) and 2.8 (20k EVs).

For the UC and UF purifications, BP dye control results were the most promising (Table 2). Unfortunately, UC did not remove the unbound BP from the labelled EVs efficiently enough during one centrifugation round: 17% (110k EVs) or 7% (20k EVs) of the dye was recovered while only 8% or less than 1% (respectively) of the EVs were collected after the centrifugation. UF gave slightly better results in terms of *E*_rp_; however, *E*_rp_ < 1 suggests a poor separation of the labelled EVs from the unbound dye, and the EV recoveries were very low (1–2%). For BP, the best purification method was UCG. The BP-labelling did not reduce the EV recoveries compared to the unlabelled controls, while majority of the fluorescent dye added to the EVs was removed during the purification. The UCG-purified BP EVs had the highest relative purification efficiencies of the studied samples, 12.7 (110k EVs) and 3.5 (20k EVs).

#### 3.3.3 BPC12- and DiO-labelled EVs

**Fig. 4:**
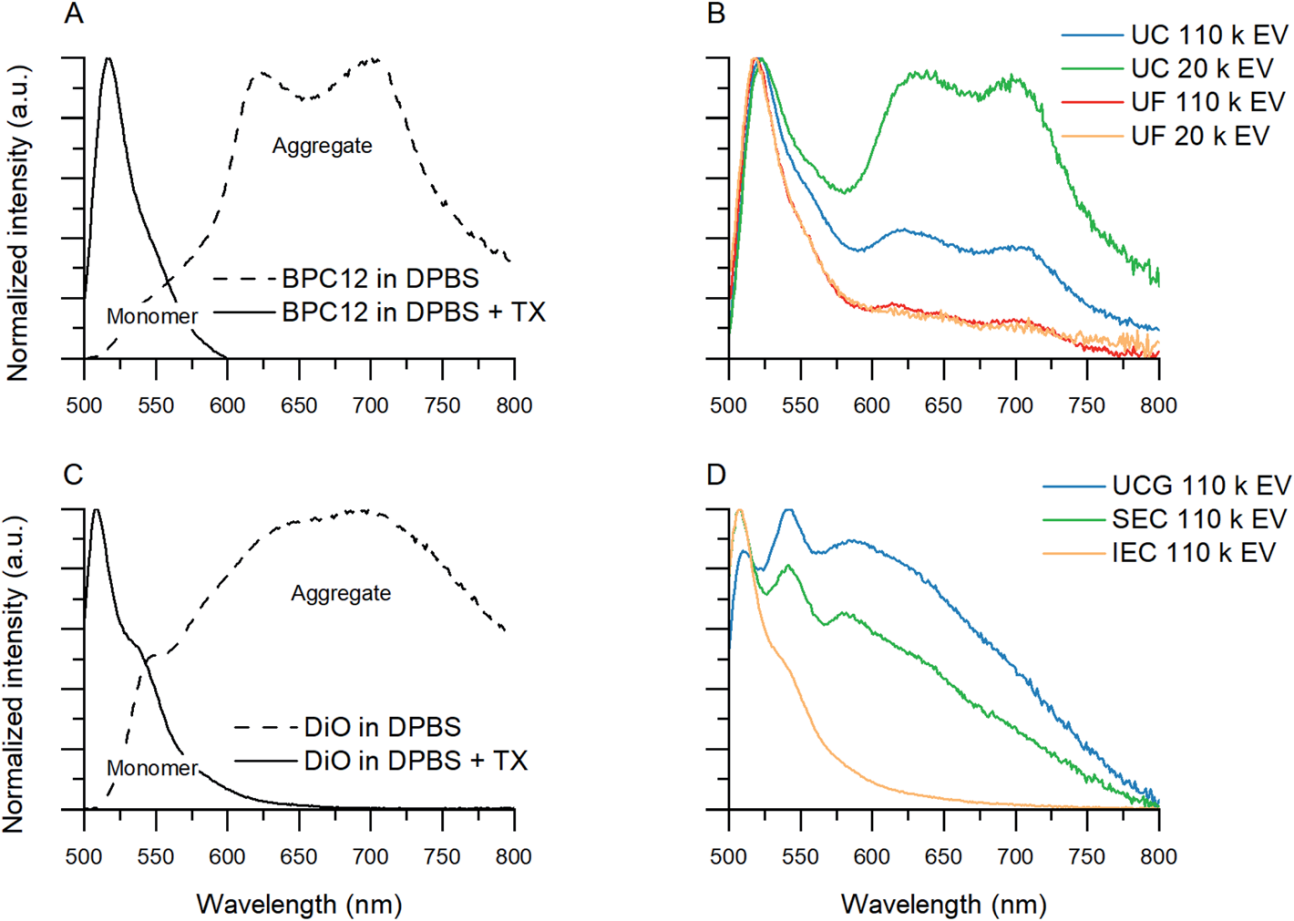
Normalized fluorescence spectra showing aggregated and monomeric BPC12 and DiO emission in solvent samples and purified EV samples. (A) Aggregated BPC12 (maxima at 630 nm and 700 nm) in DPBS and monomeric BPC12 (520 nm) in DPBS with Triton X (TX). (B) Examples of emission spectra of BPC12-labelled EVs after UC and UF purifications. (C) Aggregated DiO (broad band above 550 nm) in DPBS and monomeric DiO (maximum at 510 nm) in DPBS with TX. (D) Examples of emission spectra of DiO-labelled EVs after UCG, SEC and AEC purifications. All the spectra were measured with excitation at 483 nm.

The fluorescence spectra of the BPC12- and DiO-labelled EVs after purification could be used to evaluate the purification result as in the course of the study, the fluorescence signals of these dyes were found to be solvent-sensitive (Fig. 4A and C). When dissolved in an aqueous buffer (DPBS) with surfactant treatment, the emission spectrum of these dyes is narrow, having the maximum at 520 nm for BPC12 and 510 nm for DiO. This corresponds to the monomeric dye fluorescence. In DPBS without surfactants, the dyes are in an aggregated state, their emission is shifted to longer wavelengths (>550 nm), and the peaks are broadened. This behaviour has been reported previously for several BODIPY derivatives.^43,44^ The emission quantum yield for monomers of both dyes is much higher than that of the aggregates, and the spectra are therefore presented in a normalized form to visualise their shape. The solvent-sensitivity of the dyes is a useful property in the context of this study, as it could be used for a direct comparison of the purification results of the BPC12- and DiO-labelled EVs.

As the unbound BPC12 forms aggregates in DPBS that have similar size as the EVs (Section 3.2.2, ESI Fig. S4†), some of the particles detected by NTA are probably not EVs, and consequently *R*_EV_ is not reliable for the BPC12-labelled EVs leading to a low *E*_rp_ showing a failure of the purification. For the UC purified BPC12 EVs, a strong aggregate fluorescence was observed (Fig. 4B, green and blue). The 520 nm emission peak relates to the monomeric dye in the EV membrane, while the longer wavelength emission (bands at 630 nm and 700 nm) relates to the dye aggregates in the aqueous buffer. The relative intensity of the aggregate peaks compared to monomer (EV-related) peak is higher for 110k than 20k EVs, indicating higher concentration of the unbound BPC12 in 110k EV sample than in 20k EVs. The fluorescence spectra of the UF-purified BPC12-labelled EVs (Fig. 4B, red and yellow) do not have as pronounced aggregate emission peaks as the UC-purified EVs, indicating more efficient removal of the unbound dye especially for the 20k EVs. However, the UF purification results had high variation, which is seen in the fluorescence spectra (ESI Fig. S6†) and is reflected also in *R*_EV_ and *R*_dye_ (Table 3), suggesting that the fluorescence spectra should always be checked after the UF purification.

For DiO, UCG, SEC and AEC were studied to purify the labelled EVs. In UCG, the DiO control result showed only slight accumulation of the dye in the expected EV-fractions, while with the EVs, the dye accumulated almost exclusively in the EV-fractions (Fig. 3C, pink). This could designate strong binding of DiO to the EVs. However, given the high excess of the dye used for the labelling, there must be unbound dye present in the EV fraction. The result indicates that the EVs have affected the DiO aggregation or the diffusion of the DiO aggregates in the gradient, leading to no separation between the unbound dye aggregates and the EVs. Indeed, the fluorescence spectra of the UCG-purified DiO-labelled EVs (Fig. 4D, blue) confirms the presence of DiO aggregates in the EV fractions: monomer DiO peak at 510 nm is weak, and the spectrum is dominated by an aggregated DiO emission (bands above 550 nm). Compared to UCG, the SEC purification removed the unbound dye more efficiently. In the emission spectra of DiO EVs after SEC purification (Fig. 4D, green) the most intensive emission peak at 510 nm corresponds to the monomeric, EV-bound DiO. Nevertheless, there is still clearly a visible aggregate emission band above 550 nm. Additionally, the DiO aggregation seemed to lead to very low EV recoveries which varied from non-detectable to slightly over 1% (Table 3, ESI Table S2†).

The third method studied for purification of the DiO-labelled EVs was AEC. Similar to the SEC dye control, DiO did not pass the AEC column. However, the DiO-labelled EVs passed the AEC column better than the SEC column: the average EV recoveries were 10% for 110k EVs and 6% for 20k EVs. The fluorescence spectrum (Fig. 4D, yellow) shows only a monomeric EV-related emission peak at 510 nm, which is in line with *E*_rp_ ≥ 3.5 for both EV types, indicating AEC as the best purification method for DiO-labelled EVs.

### 3.4 FLIM imaging of purified EVs with cells

**Fig. 5:**
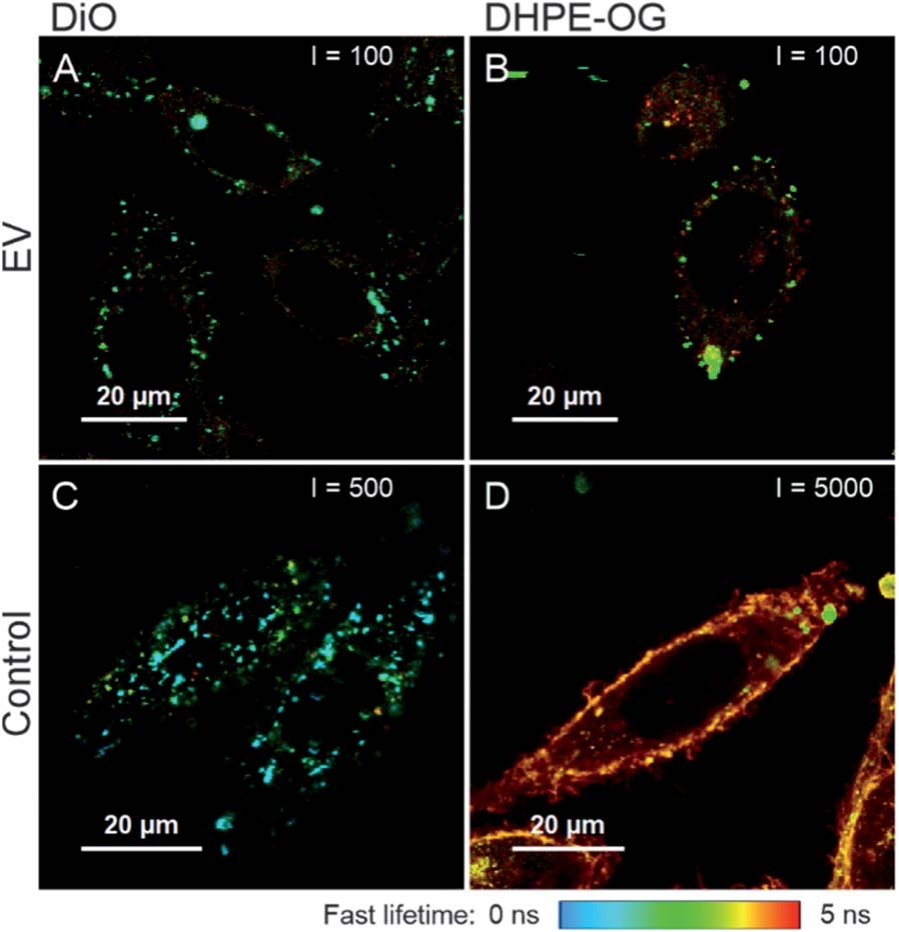
FLIM images of labelled and purified EVs (A, B) and corresponding free dyes (C and D) incubated with PC-3 cells for 3 hours. (A) AEC DiO 110k EVs, 80 000 EVs/cell; (B) DHPE-OG SEC 110k EVs, 80 000 EVs/cell; (C) DiO; and (D) DHPE-OG. The dye concentration in the free dye controls is the same as would be added with 30 000 of similar EVs/cell. The maximum intensity threshold (events) is presented in the upper corner of each figure. The figures are presented in the same fluorescence lifetime scale presented below the figure.

A selection of labelled and purified EVs and corresponding free dyes were applied to PC-3 cells and imaged with FLIM to confirm the visibility and examine the distribution of the labelled EVs in the cells, and to compare the fluorescence staining patterns to those of the free dyes. Examples of the FLIM images are presented in Fig. 5.

The FLIM imaging results demonstrate the importance of the purification step and underlines the difficulties in separating the free dye background from the fluorescence signal originating from the EVs. In general, the fluorescence intensities of the samples incubated with labelled and successfully purified EVs (Fig. 5A and B) were lower than the free dye controls (Fig. 5C and D), although the dye concentrations were higher in the EV samples, indicating slower dye internalization *via* EVs. The average fluorescence lifetime of DiO is similar in both samples (*τ*_av_ = 1.17 ns in the EV sample and *τ*_av_ = 1.16 ns in the control), while there is a clear difference in the DHPE-OG lifetimes (*τ*_av_ = 2.63 in the EV sample and *τ*_av_ = 3.99 ns in the control).

Based on *E*_rp_ (Table 3), purification of DiO and DHPE-OG 110k EVs succeeded with AEC and SEC, correspondingly. The staining patterns of the EVs labelled with these dyes are spot-like inside the cells (Fig. 5A and B), which could be interpreted as the internalization of EVs into the endosomal pathway. However, the free DiO staining pattern and fluorescence lifetime is very similar to the DiO-labelled EVs (Fig. 5A and C); consequently, it is very easy to misinterpret the free DiO background as labelled EVs. For DHPE-OG, the difference in the staining patterns and fluorescence lifetime is clearer (Fig. 5B and D), indicating different uptake mechanisms of the free dye and EV-bound dye. Free DHPE-OG seems to stay attached to the cell membrane at least for 3 h of incubation with cells while EV-bound dye enters the cell confirming the EV mediated dye internalisation. However, even if the labelling and purification of the EVs from the unbound dye would have been successful, the lipophilic dyes may leach from the EVs and stain other cellular membranes.^28,45^

Our results also demonstrate that with a successful removal of the unbound dye, the EVs might not be detectable when applied to the cells. The emission intensity of the cells incubated with SEC-purified Ptx-OG EVs (30 000 EVs/cell) was close to the cell autofluorescence, and due to low EV recoveries, intensity could not be increased by adding more EVs. The UCG-purified BP EVs gave best *E*_rp_ values but were not visible even with 400 000 EVs/cell, suggesting that either the labelling efficiency has not been high enough for detecting the EVs or the iodixanol has a negative effect on the EV-cell interactions.

## 4 Discussion

The FLIM images of the labelled EVs (Fig. 5) clearly demonstrate that although fluorescence signal is observed, it is not necessarily related to the EVs. Thus, for further reliable applications, *e.g.* in microscopy, it is extremely important to ensure both successful labelling and purification from the unbound dye with a dedicated method. To evaluate the purity of labelled EVs, we relied on a simple parameter, *E*_rp_ to describe the approximate success of each purification protocol. The approach is simple and applies to the cases where the unbound dye is present in the system after labelling or the dye-to-EV ratio is high enough. As described earlier, this is very often the case for passive and covalent labelling. Moreover, it is clear that using a dye control solely is not sufficient to validate the purification success. The unbound dye behaviour may be affected by the presence of EVs as, for example, in the case of DIO in ultracentrifugation with gradient (Fig. 3 A and C).

As the EV samples were initially isolated with a differential ultracentrifugation protocol prior to a density gradient, it may have also affected the EVs morphologically causing EV aggregation and shape distortion. The effect on the results presented in this study is difficult to estimate. However, the study is focused on screening purification methods, and the important point is that the initial EVs have been treated in the same way for all experiments.

Nonlabelled EV controls of a purification method are important as some of the methods themselves may result in almost complete EV loss. This makes the method uninteresting for labelled EV purification as in the case of consequent UCG and UF (Table 1). The comparison of nonlabelled and labelled EV recoveries also gives important information about the possible effects of the dye on EV recovery. Significant reduction of EV recovery due to labelling questions the probe's suitability for EVs. Lastly, the parameter *E*_rp_, compares EV and dye recoveries after purification. If the percentage of EV losses is higher than that of dye removal (*E*_rp_ < 1) the method obviously does not perform as desired and indicates a significant amount of unbound dye in the final labelled EVs. The need to compare the EV recovery with the dye recovery becomes clear upon taking a look at Table 3, where a relatively good amount of recovered particles in EV samples does not always match with efficient dye removal. Thus, it turns out that only the combination of the controls together with the *E*_rp_ metric leads to a comprehensive assessment of the tested purification method.

In this study, we relied on NTA for evaluating the EV recovery, assuming that labelling does not significantly change the refractive index or size of the labelled EVs, excluding EV aggregation. Due to NTA's limits, not all the smallest EVs can be detected, and the method does not discriminate between EVs and other nanoparticles. However, the use of NTA in our attempt to suggest a protocol for evaluation of purification efficiency can be justified as we compare initially well-purified EVs using the same NTA settings and the same instrument prior and after labelling and purification. Thus, the relative values, such as ratios of the concentrations measured by NTA, can be reasonable estimates of the EV recoveries. Moreover, *E*_rp_ parameter represents a criterium of a purification success, *i.e.* can reflect both successful (>1) or non-successful (<1) purification and should not be considered as absolute value in contrast to usually used labelling efficiency. The latter is meant to show a particular ratio of labels attached to a labelling target and is limited by 100% over which it becomes irrelevant.

Based on the above methodology (Scheme 1A–C), the purification methods were classified into three categories for each dye: good, unknown, and poor, (“+”, “?”, or “−“) as presented in Table 4. Most of the methods were directly classified based on the negative dye control results, and the rest were classified according to the relative purification efficiencies (*E*_rp_) of the labelled EVs. The methods which could reproducibly concentrate labelled EVs and remove unbound dye efficiently are marked with “+”. According to the same logic, the methods that can recover more dye than EVs, and therefore cannot provide good separation of the labelled EVs from the unbound dye, are marked with “−“. The third category, marked with “?”, had high variation in the purification results or yielded *E*_rp_ close to 1. These methods may be considered for removing these dyes, provided that an appropriate verification of the purification result is done separately for each sample. For the samples applied for live cell imaging, also the visibility of the EVs with cells is summarized in Table 4. As can be seen, Table 4 is quite empty and most attempted purification protocols failed. But encouragingly, it was possible to find a method that at least partly worked for each dye applying the approach proposed in our study.

**Table 4:** Summary of the purification and imaging results. Method marked with + had relative purification efficiency *E*_rp_ > 1, high variation in the results or *E*_rp_ ≈ 1 are marked with ?, and − signifies negative dye control result or *E*_rp_ < 1. For the samples that were imaged with FLIM, the visibility of the EVs is marked in the brackets (+: fluorescence was detected, −: no reliable fluorescence in cells)

Method	Ptx-OG	DHPE-OG	DiO	BPC12	BP
UC	−	−	−	−	−
UCG	−	?	−	−	+ (−)
UF	−	−	−	?	−
SEC	+ (−)	? (+)	−	−	−
AEC	−	−	+ (+)	−	−

Interestingly, the molecular structure of the dyes has a clear effect on the purification results. Unbound BP and DiO were effectively isolated from the EVs: BP has a rigid molecular skeleton and DiO two hydrocarbon chains anchoring it to the EV membrane during the labelling. On the other hand, the dyes with freely rotating units, OG-chromophore in DHPE-OG as well as the BP-chromophore in BPC12, showed either poor purification or high result variation. Furthermore, the dyes do not always act according to the expected purification principle. Ptx-OG and DiO (Table 2) form particle-like aggregates in aqueous environments that are large enough to be collected by centrifugation and BPC12 precipitates into the tube walls. Thus UC, the most used protocol for the EV purification,^46^ is not a suitable purification method for these dyes. BPC12 is also retained in the polyethersulfone-based filter membrane used in UF instead of passing it. In UCG, DiO and BPC12 gathered into the same layer as the EVs, probably due to aggregate formation. For SEC and AEC, DiO did not pass the columns at all.

From the studied methods, UC and UF were not efficient methods for purification of unbound dye from the labelled EVs. For UC, even with the dyes that stay mostly in the supernatant during the centrifugation, several UC concentration cycles would be necessary for efficient removal of the dye. However, since in most cases over 90% of the initial EVs were lost during a single UC run, no EVs would be left after few centrifugations. According to our results, also UF leads to very low EV recoveries and is, thus, not suitable for the unbound dye removal. The recoveries after UF might be improved by choosing different filter devices,^47^ but a comprehensive study of these matters is missing and there is no univocal consensus on the best way to purify EVs with UF.^24,47–51^ The use of UF for removal of the iodixanol proved to be the limiting step of UCG as well. In our work, we observed high initial UCG EV yields of 60–80% before gradient removal, while using the common ultrafiltration protocol for removing the iodixanol from the EVs, we observed a heavy loss of EVs down to ∼1% yield (Table 1). Despite the wide usage of UCG for EV purification, methods to remove the iodixanol are not yet fully addressed. As iodixanol is a non-charged small-sized molecule, both AEC and SEC are promising candidate methods for the iodixanol removal.

The use of chromatographic methods for EV isolation and purification has raised a lot of interest, because they can be highly automatized and scaled up.^52,53^ From the two chromatographic methods studied here, SEC showed more potential for removing the unbound dye from the labelled EVs, and is already a commonly used technique in EV field with different ready to use solutions available on the market.^54^ On the other hand, AEC was incompatible with the studied dyes, although the unlabelled EV recoveries were high. DiO-labelled EVs were successfully purified with AEC; however, the purification principle was rather related to the dye aggregation. With both chromatographic methods, the recoveries of labelled EVs were smaller than those of non-labelled EVs, suggesting that the dye may partly clog the column or increase EV binding to the column. The AEC method provided an interesting comparison for SEC as the EV losses were smaller in AEC than in SEC. This can be at least partially explained by non-specific binding of EVs to the column: the SEC bed size was more than 10-fold larger than the AEC bed size, therefore containing much more surface area for binding. Furthermore, saturating the AEC column with BSA significantly increased the EV recoveries. The results indicate that the EV yields could be increased in the SEC by reducing the column volume and if feasible, by blocking the column with BSA.

Based on this study, it is difficult to say whether the dye is actually located in the EV membrane. Still, *E*_rp_ is a useful tool for the pre-screening of the suitable dye-purification method combinations. As some dyes, such as PKH dyes^24^ or BPC12 used here, tend to form dye aggregates with similar sizes as the EVs, detecting fluorescent EV-sized particles is not sufficient proof for the successful EV labelling and purification and there is clearly a need for a method for separating the EVs from possible dye aggregates. Generally, EV marker antibodies are recommended for separating EVs from other particles.^38^ Consequently, quantitative particle detection methods such as NTA^12,13^ and flow cytometry^10,11^ could offer a solution for identifying the labelled EVs.

## 5 Conclusions

Five common purification methods were tested with five dyes for their ability to separate the passively labelled EVs from the unbound dye. None of the studied purification methods was suitable for all the studied dyes. Most dyes could be successfully purified with only one of the methods tested, suggesting that the successful purification method is related to the physical and chemical properties of the dye. Notably, in many cases the expected purification principle did not work. The unbound dye might form aggregates, bind to the purification matrices or gather to the same location with the EVs for some other reason. Thus, we suggest the following steps when working with a new dye to ensure the successful removal of the unbound dye: (1) studying the behaviour of free dye and unlabelled EVs separately with the chosen method to determine whether the method can effectively separate those from each other, (2) ensuring the separation still exists with the labelled EVs, and (3) evaluating the purification quality by *E*_rp_. Importantly, the labelled EVs need to have high enough fluorescence intensity to be visible in the target application that is not always the case after successful purification.

The most promising methods for the used dyes were SEC and UCG, and the highest recoveries were obtained by UCG before removing the density gradient. With SEC, the purification is faster to perform involving only one separation step, making the SEC purification favourable over the UCG when there is a possibility to choose between the methods. However, both methods should be further developed: the gradient removal step after UCG causes high EV losses and SEC purification should be still improved to yield higher EV recoveries. Sometimes also an unexpected purification principle may provide good purification results, as we demonstrated with AEC.

In this study, we used only EVs from a single cell line: the purification results with the same dyes but EVs from different sources might be different. We admit that the *E*_rp_ values used in this study to evaluate the success of the purification of labelled EVs from unbound dye is an approximate method. However, the parameter in combination with the proposed controls allows to assess the purification outcome in a more comprehensive way compared to using only dye controls or labelling efficiency values. The proposed approach represents an easy methodology for initial pre-screening of multiple labelling conditions and purification methods.

## Author contributions

Conceptualization: KR, EV-L, ESL; methodology: KR, HS, EV-L, ESL; investigation: KR, JZ, EL, HS, IH, JL, SV, AE, ESL; writing – original draft: KR, JZ, HS, AE, MY, TL, EV-L, ESL; supervision: MY, TL, EV-L, ESL.

## Conflicts of interest

There are no conflicts to declare.

## Supplementary Material

NA-004-D1NA00755F-s001
