# Correction: Addressing challenges in the removal of unbound dye from passively labelled extracellular vesicles

**DOI:** 10.1039/d1na90120f

**Published:** 2021-12-23

**Authors:** Kaisa Rautaniemi, Jacopo Zini, Emilia Löfman, Heikki Saari, Iida Haapalehto, Johanna Laukka, Sami Vesamäki, Alexander Efimov, Marjo Yliperttula, Timo Laaksonen, Elina Vuorimaa-Laukkanen, Ekaterina S. Lisitsyna

**Affiliations:** Chemistry and Advanced Materials, Faculty of Engineering and Natural Sciences, Tampere University Korkeakoulunkatu 8 33720 Tampere Finland ekaterina.lisitsyna@tuni.fi; Drug Research Program, Division of Pharmaceutical Biosciences, Faculty of Pharmacy, University of Helsinki Viikinkaari 5 00790 Helsinki Finland; Finnish Red Cross Blood Services Kivihaantie 7 00310 Helsinki Finland

## Abstract

Correction for ‘Addressing challenges in the removal of unbound dye from passively labelled extracellular vesicles’ by Kaisa Rautaniemi *et al.*, *Nanoscale Adv.*, 2022, DOI: 10.1039/d1na00755f.

The authors regret that an incorrect version of Table 3 was included in the original article. The correct version is given here:

**Table:** EV recoveries *R*_EV_, dye recoveries in the EV fractions *R*_dye_, and relative purification efficiencies *E*_rp_ for the labelled and purified EVs. The removal of unbound dye was studied with ultracentrifugation (UC), ultracentrifugation with density gradient without ultrafiltration (UCG), ultrafiltration (UF), size-exclusion chromatography (SEC), and anion exchange chromatography (AEC). The individual values for each replicate are presented in ESI Table S2

Dye	Method	110k EVs			20k EVs		
		*R*_EV_ a (%)	*R*_dye_ (%)	*E*_rp_ a ^,^ b	*R*_EV_ a (%)	*R*_dye_ (%)	*E*_rp_ a ^,^ b
DHPE-OG	UCG	43.0 ± 2.8†	44.6 ± 4.2	1.0	52.9 ± 7.5†	39.6 ± 3.3	1.3
	SEC	12.2 ± 1.6†	8.7 ± 1.4	1.4	8.2 ± 0.9	9.3 ± 5.0	0.9
Ptx-OG	UCG	10.3 ± 0.4†	67.8 ± 11.5	0.2‡	6.5 ± 3.3	41.3 ± 38.4	0.2‡
	SEC	3.8 ± 1.6	2.9 ± 1.3	1.3	3.7 ± 0.9	1.3 ± 0.3	2.8†
BP	UC	7.6 ± 4.8	16.6 ± 1.3	0.5‡	<1 ‡	7.0 ± 0.8	—
	UF	1.2 ± 0.7‡	1.8 ± 0.9	0.7‡	2.3 ± 1.0‡	4.8 ± 3.8	0.5‡
	UCG	78.6 ± 10.3†	6.2 ± 0.9	12.7†	54.0 ± 6.0†	15.5 ± 4.8	3.5†
BPC12	UC	12.5 ± 6.8†	35.5 ± 37.6	0.4‡	5.9 ± 6.2	17.2 ± 15.3	0.3‡
	UF	3.9 ± 2.8	7.9 ± 3.4	0.5‡	8.4 ± 11.2	9,5 ± 15,4	0.9‡
DiO	UCG	n.d.	n.d.	—	n.d.	n.d	—
	SEC	1.1 ± 0.2‡	n.d.	—	<1‡	n.d	—
	AEC	10.1 ± 12.3†	2.2 ± 2.0	4.6†	6.4 ± 0.3	1.8 ± 0.3	3.5†

a † – acceptably high; ‡ – unacceptably low; all other values are acceptable with caution.

b *E*_rp_ > 1 indicates successful separation of the labelled EVs from the unbound dye: the greater *E*_rp_, the better separation; conversely, *E*_rp_ < 1 indicates unsuccessful removal of the dye.

The Royal Society of Chemistry apologises for these errors and any consequent inconvenience to authors and readers.

## Supplementary Material

