# Supplementary material for: Addressing challenges in the removal of unbound dye from passively labelled extracellular vesicles
Source: Nanoscale Adv. 2021 Nov 23;4(1):226–40. doi: 10.1039/d1na00755f (PMC9417910; doi:10.1039/d1na00755f)
Supplement: NA-004-D1NA00755F-s001 [file NA-004-D1NA00755F-s001.pdf]

Electronic Supplementary Material (ESI) for Nanoscale Advances.
This journal is © The Royal Society of Chemistry 2021

Supplementary information

Addressing challenges in the removal of unbound dye from passively labelled extracellular vesicles

Supplementary section S1. Characterization of the isolated EVs

S1.1 Protein assays

Protein concentration of the isolated EVs was determined by bicinchoninic acid assay according to the manufacture protocol (BCA Protein Assay Kit, Millipore, USA). Each sample was analysed in triplicate. Then, the samples were analysed by Western blot. 20 µg of protein and cell lysate were incubated under reducing or non-reducing conditions at 95 °C for 15 min, loaded in Mini-PROTEAN TGX™ gels (Bio-Rad, USA) 4-20 %, and, after electrophoresis, transferred in poly(vinylidene fluoride) (PVDF, Bio-Rad) membrane. The PVDF membrane was then blocked with 3 % (w/v) bovine serum albumin (BSA) in Tris-buffered saline-0.1% (v/v) Tween 20 (TBS-T) 1 h at RT and cut in 5 strips based on the molecular weight of the protein marker of interest. The membrane strips were then incubated with the respective antibodies: monoclonal anti-GM130 (1:250, reducing conditions, isotype: rabbit, cat. code: NBP1-89756, Novus Biologicals, USA), anti-Hsp70 (1:1000, reducing conditions, clone: 7/Hsp70, isotype: mouse IgG1, cat. code: 610607, BD Transduction Laboratories, USA), anti α-Tubulin (1:1500, reducing conditions, clone: 6A 204, isotype: mouse IgG1, BP Transduction Laboratories, USA), anti-TSG101 (1:250, reducing conditions, clone: 51/TSG101, isotype: mouse IgG1, cat. code: 612697 BD Transduction Laboratories), and anti CD9 (1:1000, non-reducing conditions, clone: ALB 6, isotype mouse IgG1, cat. code: HBM-CD9, Hansa Bio Med, Estonia) in 3 % BSA in TBS-T at RT. The excess of antibodies was removed by 4 washes with TBS-T. Then, CD9, Tubulin, Hsp70 and TSG101 strips were incubated for 1 h at RT with goat anti-mouse IgG-HRP secondary antibody (1:4 000, Polyclonal, isotype: Goat IgG, cat. code: PA1-74421, ThermoFisher Scientific, USA) and GM130 strip with goat anti-rabbit IgG

GM130 (1:10 000, cat. code: G-21234, ThermoFisher Scientific, USA). The strips where washed 3 times with TBS-T and briefly with TBS, then incubated in ECL substrate (Clarity™ Western ECL Substrate, BioRad, USA) and imaged with ChemiDoc MP Imaging System (Bio-Rad, California, US). Image processing and signal intensity was assessed with ImageJ software.

S1.2 Transmission electron microscopy

EVs were prepared for the transmission electron microscopy (TEM) as described in the reference [1] by loading to carbon coated and glow discharged 200 mesh copper grids with pioloform support membrane. Briefly, EVs were fixed with 2.0 % PFA in $NaPO_4$ buffer, stained with 2 % neutral uranyl acetate, further stained and embedded in uranyl acetate and methyl cellulose mixture (1.8 / 0.4 %). Then, the EVs were viewed with TEM using Jeol JEM-1400 (Jeol Ltd., Japan) microscope operating at 80 kV. Images were taken with Gatan Orius SC 1000B CCD-camera (Gatan Inc., USA) with 4008 × 2672 pixel image size and no binning.

S1.3 Attenuated total reflection Fourier transfer infrared spectrophotometer (ATR-FTIR)

IR spectra were measured with ATR-FTIR (Spectrum One spectrophotometer, Perkin Elmer Inc., USA). 8 µl of the EV suspension (~ 5×10^{11} particles/ml) was placed over the ATR crystal and dried with airflow in order to obtain a thin layer of EVs, which covered the entire crystal surface. Measurements were performed at RT with 32 scans with a nominal resolution of 4 cm^{-1}. Raw data were elaborated as following: 1) DPBS background subtraction, 2) baseline correction, and 3) normalization based on the amide I peak. The data manipulation is presented in **Supplementary Figure S1**.

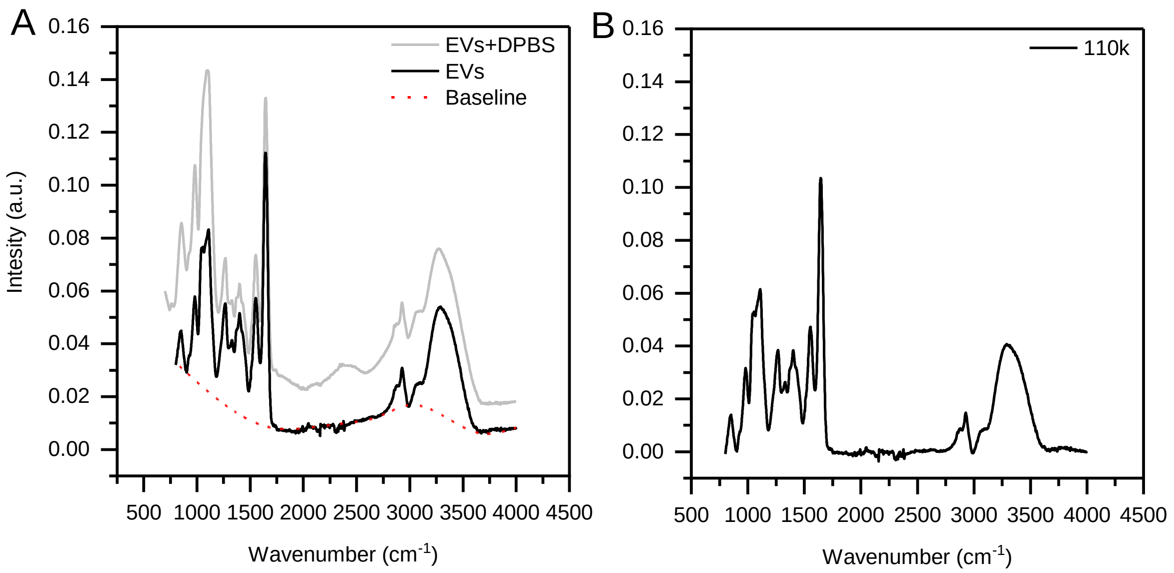

Supplementary Figure S1. The manipulation of the ATR-FTIR spectra. A) The ATR-FTIR raw spectrum of 110 k EVs (grey) and the same spectrum after subtracting the DPBS spectrum (black). The baseline is displayed in red. B) The ATR-FTIR spectrum of 110 k EVs after the baseline subtraction.

S1.4 EV characterization results

TEM picture of all samples presented EVs of typical morphology and variable sizes, with the 20 k samples having a higher proportion of large EVs (**Supplementary Figure S2A**). The NTA results underline a clear difference in size when the particle size mode and mean are compared (**Supplementary Figure S2B**), as 20 k EVs are significantly larger than 110 k EVs. Particle size distribution (**Supplementary Figure S3**) also presents a shift towards a bigger size of 20 k EVs compared to the 110 k EVs.

Protein markers for western blotting were chosen based on the MISEV2018 guidelines [2]: Hsp70 and TSG101 are cytosolic proteins often enriched in EVs, CD9 is a transmembrane protein mainly associated to small EVs, and GM130 is localized to the Golgi apparatus. The presence of GM130 is associated with cellular debris and therefore indicates inefficient EV purification. The WB results (**Supplementary Figure S2C**) demonstrate the enrichment of the three EV markers (Hsp70, TSG101 and CD9) in both EV subpopulations, especially in the 110 k EVs, while the negative control (GM130) is absent from both of the EV preparations,

showing that EVs have been successfully separated from cellular contaminants. α-Tubulin, used as loading control, is present in all the blots underlining a homogenous loading of material.

Lastly, information on the molecular composition and relative amounts of biomolecules are displayed by the FTIR spectra (**Supplementary Figure S2D**): two intense amide peaks at 1644 cm^{-1} (amide I) and at 1555 cm^{-1} (amide II), originating from C=O stretching vibration and N-H bending vibration, respectively. The bands at 2879 and 2930 cm^{-1} are attributed to the symmetric and antisymmetric CH_2 groups, with the latter being more intense. The fingerprint region (600-1400 cm^{-1}) shows a strong correlation between the 20 k and 110 k EVs subpopulations. Based on the FTIR analysis, the 20 k and 110 k EVs are very similar in their composition and have similar protein to lipid ratios. The main difference can be observed between 1040 and 1110 cm^{-1} where 20 k EVs have two close peaks that overlap in the 110 k EV spectrum. However, 1040 cm^{-1} and 1110 cm^{-1} peaks fall in the fingerprint area, thus, it is difficult to associate them univocally to any functional group.

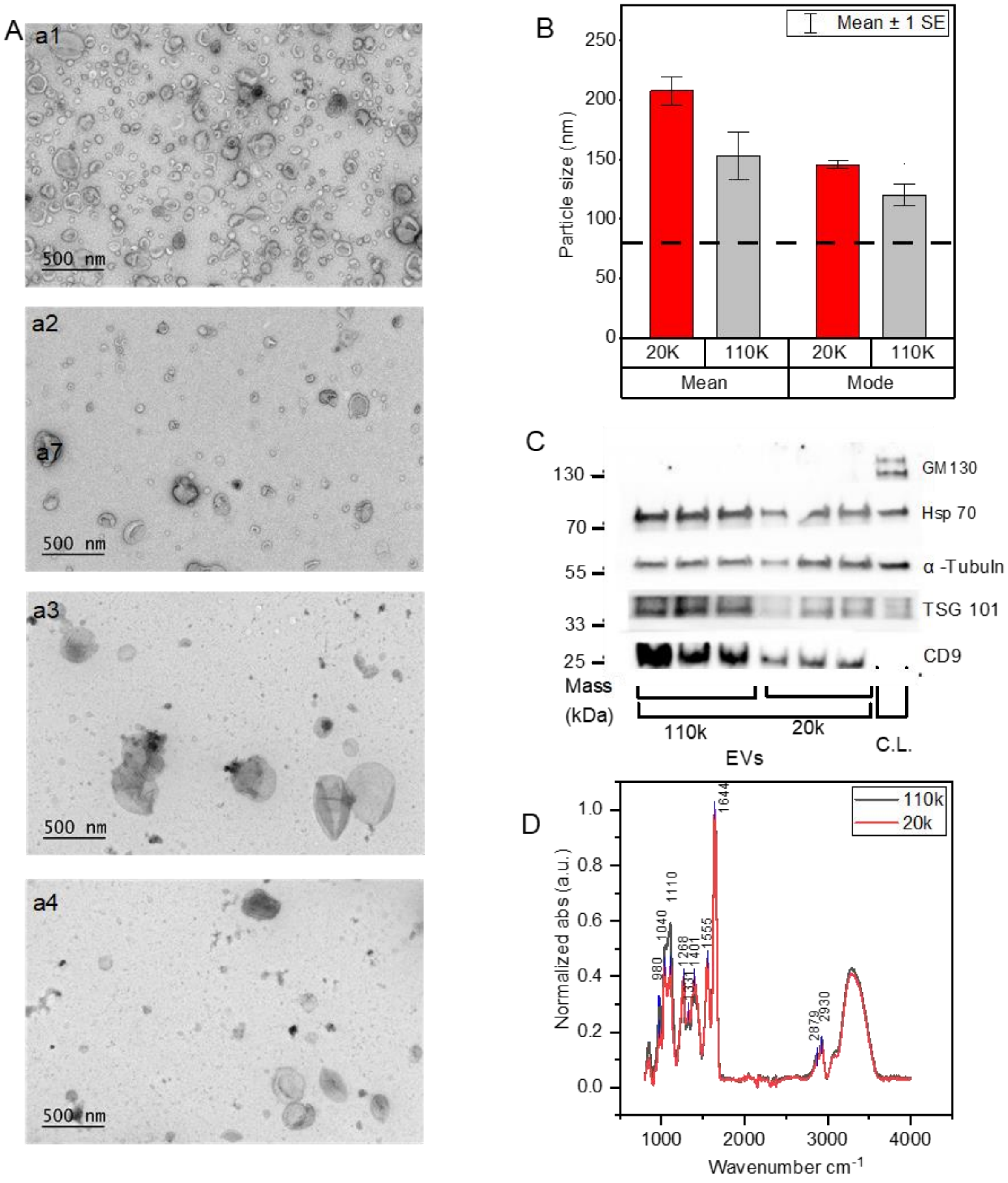

Supplementary Figure S2. Characterization of EVs by transmission electron microscope, NTA, Western blot and FTIR. A) Electron micrographs of 110 k (a1-a4) and 20 k (a5-a8) EVs. B) Mean and mode of 20 k and 110 k EVs determined by NTA ± standard error, dashed line – NTA detection limit 80 nm. C) Western blot analysis of EV markers. Hsp70, TSG 101 and CD9 are enriched in the EVs compared to the housekeeping protein α-Tubulin, which is used as a loading control. Negative control GM130 is only visible in the cell lysate, indicating the absence of cellular contaminants in the isolated EVs. Triplicates of 20 k and 110 k EVs were analysed with the cell lysate, loading 20 µg of protein per lane. D) IR spectra of 20 k and 110 k EV pellets.

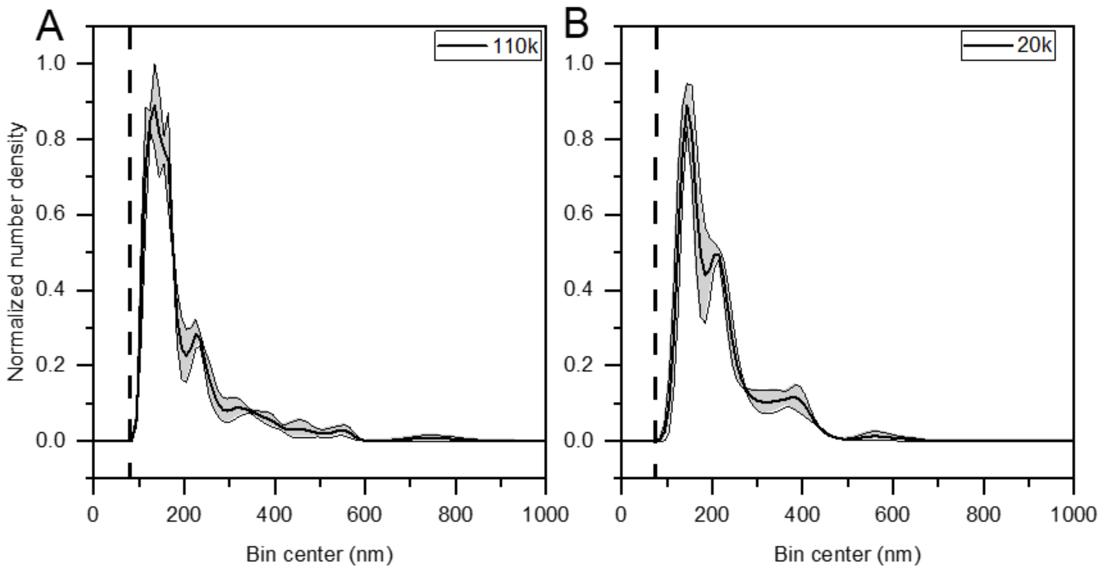

Supplementary Figure S3. Particle size distribution obtained by NTA for 110 k EVs (A) and 20 k EVs (B). The grey area represents the standard error, N=3. Dashed line represents NTA detection limit (80 nm).

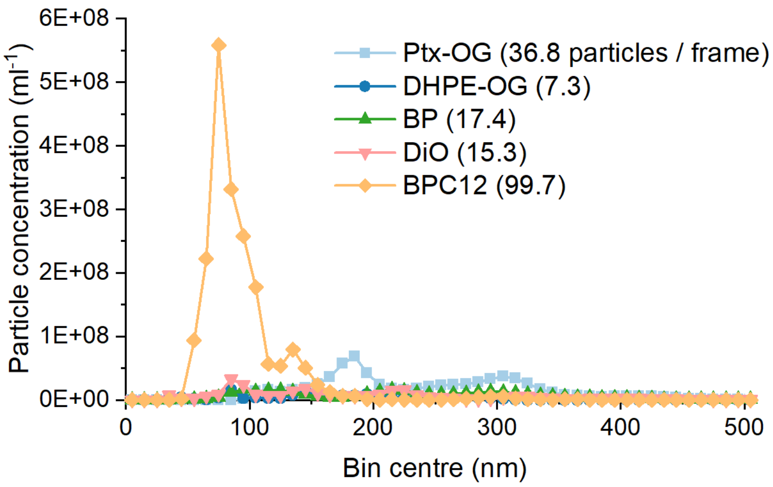

Supplementary Figure S4. NTA size distributions for 1 µM of dye in DPBS. The dye was mixed with DPBS and mixed with a vortex-mixer briefly before the NTA measurement. For Ptx-OG, the camera level was set to 10 instead of 15 used for other samples due to high scattering from the dye and therefore it is not directly comparable with the other distributions.

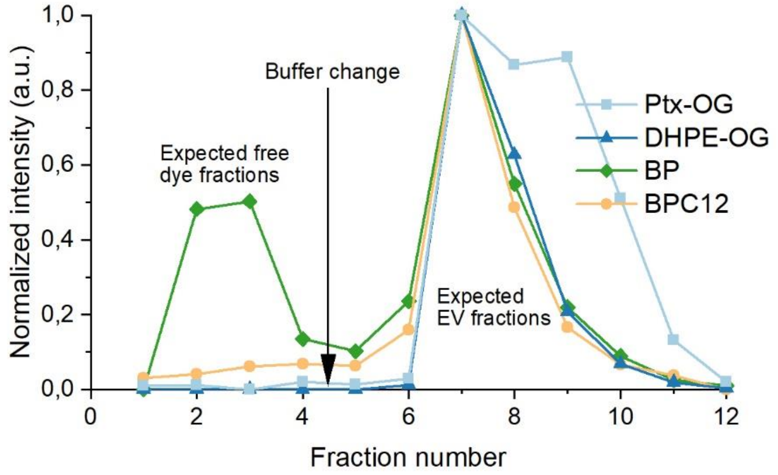

Supplementary Figure S5. Dye control results for the AEC. Normalized fluorescence intensity is presented as a function of 0.5 ml fraction number. DiO did not pass the AEC column in sufficient amounts for a reliable measurement and is therefore not shown here. Majority of the rest of the dyes eluted in the same fractions as EVs in the EV controls (fractions 6-12 with peak concentration in fraction 7).

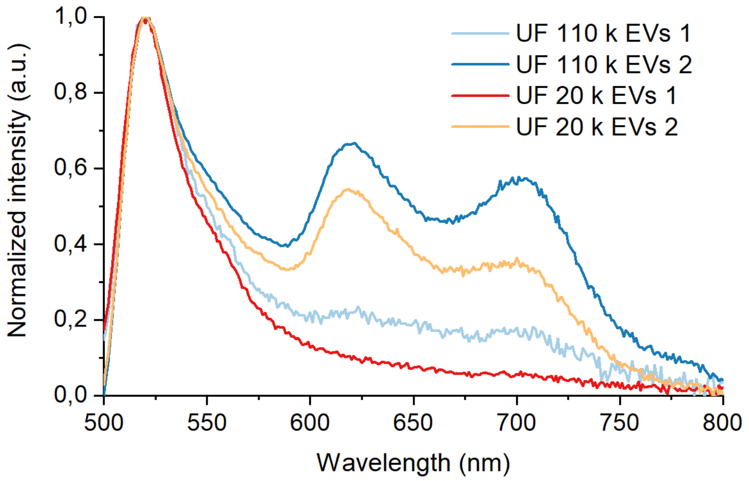

Supplementary Figure S6. Examples of fluorescence spectra of UF-purified BPC12 EVs. The spectra show high variation in the purification results between parallel samples: in replicate 1, almost no free dye background (emission bands above 600 nm) is detected, while in replicate 2 the dye background is clearly visible.

Supplementary Table S1. Individual recoveries of non-labelled EVs R_{EV} for all replicates after different purification methods: ultracentrifugation (UC), ultracentrifugation with density gradient without ultrafiltration (UCG) and with ultrafiltration (UCG + UF), ultrafiltration (UF), size-exclusion chromatography (SEC), and anion exchange chromatography (AEC).

Method	110 k R_{EV} (%)	20 k R_{EV} (%)
UC	9.2	15.0
	12.4	36.1
	15.1	20.7
UCG	69.0	65.3
	87.8	60.1
	90.1	53.0
UCG + UF	0.5	1.0
	0.5	1.4
	0.6	1.4
UF	7.8	28.6
	1.5	2.1
	3.7	2.7
SEC	52.6	16.7
	25.9	8.4
	22.9	5.4
AEC	58.1	56.3
	64.2	39.6
	71.8	42.0

Supplementary Table S2. Individual EV recoveries R_{EV}, dye recoveries R_{dye}, and relative purification efficiencies E_{rp} for the labelled and purified EVs for all replicates. The removal of unbound dye was studied with ultracentrifugation (UC), ultracentrifugation with density gradient (UCG), ultrafiltration (UF), size-exclusion chromatography (SEC), and anion exchange chromatography (AEC).

		**110 k EVs**			**20 k EVs**		
**Dye**	**Method**	R_{EV} **(%)**	R_{dye} **(%)**	E_{rp} **(%)**	R_{EV} **(%)**	R_{dye} **(%)**	E_{rp} **(%)**
DHPE-OG	UCG	44.86	44.25	1.01	45.67	39.6	1.15
		44.37	48.84	0.91	52.38	36.36	1.44
		39.80	40.57	0.98	60.60	42.82	1.42
	SEC	13.10	8.51	1.54	8.09	6.16	1.31
		13.10	10.07	1.30	9.08	6.68	1.36
		10.42	7.42	1.40	7.40	15.02	0.49
Ptx-OG	UCG	10.12	54.55	0.19	4.61	41.16	0.11
		10.62	73.91	0.14	4.52	3.05	1.48
		10.02	74.80	0.13	10.28	79.70	0.13
	SEC	4.99	3.12	1.60	4.45	1.62	2.75
		2.04	1.60	1.28	3.97	1.16	3.42
		4.34	4.09	1.06	2.73	1.16	2.35
BP	UC	13.13	17.78	0.74	0.51	7.02	0.07
		5.06	16.64	0.30	0.25	6.24	0.04
		4.70	15.33	0.31	0.25	7.81	0.03
	UF	2.00	2.82	0.71	1.40	0.61	2.30
		1.01	1.05	0.96	3.29	7.97	0.41
		0.62	1.63	0.38	2.08	5.80	0.36
	UCG	69.89	5.87	11.91	54.99	13.11	4.19
		75.91	5.61	13.53	59.29	21.02	2.82
		89.93	7.13	12.61	47.58	12.37	3.85
BPC12	UC	20.15	78.53	0.26	13.05	34.82	0.37
		7.09	18.57	0.38	2.08	8.16	0.25
		10.32	9.24	1.12	2.69	8.51	0.32
	UF	2.61	8.69	0.30	21.26	27.23	0.78
		7.08	10.71	0.66	1.76	0.43	4.09
		2.08	4.14	0.50	2.26	0.71	3.18

Continues in the next page

Supplementary Table S2 continues

		110 k EVs			20 k EVs		
**Dye**	**Method**	R_{EV} **(%)**	R_{dye} **(%)**	E_{rp} **(%)**	R_{EV} **(%)**	R_{dye} **(%)**	E_{rp} **(%)**
DiO	UCG	n.d.	n.d.	-	n.d.	n.d.	-
	SEC	0.99	n.d.	-	not detected	n.d.	-
		1.10	n.d.	-	not detected	n.d.	-
		1.30	n.d.	-	0.22	n.d.	-
	AEC	4.03	1.09	3.70	6.21	2.09	2.97
		24.27	4.58	5.30	6.69	1.65	4.05
		2.05	0.96	2.14	6.26	1.73	3.62

Supplementary Table S3. NTA results used for calculation of R_{EV} values for labelled EVs after purification (Table S2).

**Dye**	**Method**	**EV sample**	**Concentration, particles/ml**	**Number of particles detected per frame**	**replicate #**
DHPE-OG	UCG	110 k EVs	fr 8: 4.52×10^{10}	91.8 ± 2.6	1
			fr 8: 4.47×10^{10}	90.7 ± 1.4	2
			fr 8: 4.01 x 10^{10}	81.4 ± 6.0	3
		20 k EVs	fr 8: 4.56×10^{10}	46.3 ± 2.3	1
			fr 8: 5.23×10^{10}	53.1 ± 3.6	2
			fr 8: 6.04 x 10^{10}	61.3 ± 6.8	3
	SEC	110 k EVs	fr 4: 7.8×10^{9}<br>fr 5: 5.4×10^{9}	58.2 ± 6.3<br>27.4 ± 1.6	1
			fr 4: 6.5×10^{9}<br>fr 5: 6.7×10^{9}	16.5 ± 0.7<br>27.4 ± 1.6	2
			fr 4: 7.9×10^{9}<br>fr 5: 2.6×10^{9}	40.1 ± 3.3<br>13.4 ± 0.6	3
		20 k EVs	fr 4: 2.0×10^{9}<br>fr 5: 6.2×10^{9}	20.6 ± 3.2<br>31.2 ± 1.6	1
			fr 4: 4.7×10^{9}<br>fr 5: 4.5×10^{9}	47.9 ± 10.0<br>45.4 ± 7.7	2
			fr 4: 5.1×10^{9}<br>fr 5: 2.4×10^{9}	26.1 ± 2.2<br>24.7 ± 0.5	3

Continues in the next page

Supplementary Table S3 continues

Dye	Method	EV sample	Concentration, particles/ml	Number of particles detected per frame	replicate #
Ptx-OG	UCG	110 k EVs	fr 8: 1.02×10^{10}	20.7 ± 0.6	1
			fr 8: 1.07×10^{10}	21.7 ± 1.8	2
			fr 8: 1.01 x 10^{10}	20.4 ± 0.8	3
		20 k EVs	fr 8: 2.75×10^{9}<br>fr 9: 2.23×10^{9}	27.9 ± 4.5<br>4.5 ± 0.2	1
			fr 9: 4.88×10^{9}	12.4 ± 1.2	2
			fr 9: 1.11 x 10^{10}	14.0 ± 1.7	3
	SEC	110 k EVs	fr 4: 2.55×10^{9}<br>fr 5: 2.48×10^{9}	25.9 ± 1.3<br>12.6 ± 1.9	1
			fr 4: 1.47×10^{9}<br>fr 5: 5.91×10^{8}	15.0 ± 1.3<br>6.0 ± 0.4	2
			fr 4: 3.12×10^{9}<br>fr 5: 1.25×10^{9}	31.7 ± 3.3<br>12.6 ± 0.4	3
		20 k EVs	fr 4: 1.86×10^{9}<br>fr 5: 2.95×10^{9}	18.8 ± 1.5<br>29.9 ± 0.1	1
			fr 4: 1.97×10^{9}<br>fr 5: 2.32×10^{9}	20.0 ± 2.0<br>23.6 ± 2.0	2
			fr 4: 1.12×10^{9}<br>fr 5: 1.83×10^{9}	11.3 ± 0.9<br>18.6 ± 1.5	3
BP	UC	110 k EVs	1.89×10^{11}	47.8 ± 4.8	1
			7.29×10^{10}	147.9 ± 17.2	2
			6.77 x 10^{10}	114.6 ± 12.9	3
		20 k EVs	1.10×10^{10}	11.2 ± 0.8	1
			3.18×10^{9}	6.5 ± 1.8	2
			4.20 x 10^{9}	7.1 ± 0.8	3
	UF	110 k EVs	1.42×10^{10}	72.2 ± 1.2	1
			6.44×10^{9}	16.3 ± 0.5	2
			3.72 x 10^{9}	18.9 ± 2.0	3
		20 k EVs	1.06×10^{10}	53.8 ± 6.2	1
			1.18×10^{10}	59.7 ± 6.9	2
			1.06 x 10^{10}	54.0 ± 4.8	3
	UCG	110 k EVs	fr 8: 6.25×10^{10}<br>fr 9: 7.91×10^{9}	63.5 ± 5.6<br>8.0 ± 0.4	1
			fr 8: 6.70×10^{10}<br>fr 9: 9.48×10^{9}	68.0 ± 4.8<br>19.2 ± 1.5	2
			fr 8: 7.84×10^{10}<br>fr 9: 1.22×10^{10}	79.6 ± 5.9<br>24.8 ± 1.9	3
		20 k EVs	fr 8: 2.97×10^{10}<br>fr 9: 2.52×10^{10}	60.3 ± 5.6<br>25.5 ± 2.3	1
			fr 8: 2.72×10^{10}<br>fr 9: 3.20×10^{10}	55.2 ± 6.8<br>32.5 ± 2.0	2
			fr 8: 2.60×10^{10}<br>fr 9: 2.15×10^{10}	57.2 ± 4.5<br>21.8 ± 2.3	3

Continues in the next page

Supplementary Table S3 continues

Dye	Method	EV sample	Concentration, particles/ml	Number of particles detected per frame	replicate #
BPC12	UC	110 k EVs	1.08×10^{11}	118.4 ± 16.0	1
			1.04×10^{11}	26.5 ± 3.3	2
			2.67 x 10^{11}	13.6 ± 3.4	3
		20 k EVs	3.38×10^{10}	37.0 ± 4.9	1
			3.39×10^{10}	17.2 ± 2.5	2
			1.54 x 10^{11}	15.7 ± 2.2	3
	UF	110 k EVs	2.50×10^{10}	12.7 ± 1.9	1
			4.69×10^{10}	23.8 ± 5.6	2
			1.21 x 10^{10}	29.9 ± 5.2	3
		20 k EVs	6.43×10^{10}	26.1 ± 4.3	1
			6.59×10^{9}	3.3 ± 1.3	2
			1.43 x 10^{10}	23.6 ± 6.3	3
DiO	SEC	110 k EVs	fr 4: 9.91×10^{8}	10.1 ± 1.5	1
			fr 4: 5.80×10^{8}<br>fr 5: 5.18×10^{8}	5.9 ± 0.6<br>6.6 ± 1.5	2
			fr 4: 1.32×10^{9}	16.7 ± 1.9	3
		20 k EVs	n.d.	n.d.	1
			n.d.	n.d.	2
			fr 4: 6.01×10^{8}	7.6 ± 0.8	3
	AEC	110 k EVs	fr 7: 3.16×10^{9}<br>fr 8: 9.46×10^{8}	16.1 ± 1.7<br>9.6 ± 0.7	1
			fr 6: 1.13×10^{9}<br>fr 7: 5.04×10^{9}<br>fr 8: 1.89×10^{9}	12.3 ± 0.9<br>51.2 ± 3.0<br>19.2 ± 1.3	2
			fr 6: 1.49×10^{10}<br>fr 7: 2.62×10^{10}<br>fr 8: 7.44×10^{9}	?<br>?<br>?	3
		20 k EVs	fr 6: 4.05×10^{9}<br>fr 7: 5.90×10^{9}<br>fr 8: 2.47×10^{9}	20.6 ± 0.8<br>30.0 ± 1.2<br>18.7 ± 4.7	1
			fr 6: 2.52×10^{9}<br>fr 7: 8.82×10^{9}<br>fr 8: 2.04×10^{9}	12.8 ± 0.2<br>44.8 ± 4.3<br>10.3 ± 0.2	2
			fr 6: 3.75×10^{9}<br>fr 7: 8.11×10^{9}<br>fr 8: 4.87×10^{9}	?<br>?<br>?	3

Supplementary Table S4. NTA results used for calculation of R_{EV} values for non-labelled EVs (control) after purification (Tables S1)

Method	EV sample	Concentration, particles/ml	Number of particles detected per frame	replicate #
UCG (without UF)	110 k EVs	fr 8: 6.90×10^{10}	70.0 ± 4.6	1
		fr 8: 8.78×10^{10}	89.1 ± 10.9	2
		fr 8: 9.01×10^{10}	91.5± 9.9	3
	20 k EVs	fr 8: 6.53×10^{10}	66.3 ± 10.1	1
		fr 8: 6.01×10^{10}	61.0 ± 7.1	2
		fr 8: 1.03×10^{10}<br>fr 9: 4.27×10^{10}	21.0 ± 1.8<br>43.4 ± 2.1	3
UCG + UF	110 k EVs	2.89×10^{9}	14.7 ± 2.3	1
		4.00×10^{9}	40.6 ± 1.6	2
		4.60×10^{9}	23.4 ± 2.0	3
	20 k EVs	5.76×10^{9}	29.3 ± 1.4	1
		5.85×10^{9}	29.7 ± 3.6	2
		7.56×10^{9}	15.3 ± 2.6	3
UF	110 k EVs	8.27×10^{9}	42.0 ± 3.3	1
		1.56×10^{10}	79.3 ± 8.1	2
		4.51×10^{10}	22.9 ± 3.4	3
	20 k EVs	1.01×10^{10}	51.1 ± 6.2	1
		1.37×10^{10}	69.6 ± 9.2	2
		7.95×10^{10}	40.3 ± 5.6	3
UC	110 k EVs	2.14×10^{11}	54.4 ± 7.0	1
		2.61×10^{11}	66.3 ± 10.0	2
		1.01×10^{111}	51.2 ± 7.1	3
	20 k EVs	5.26×10^{11}	26.7 ± 2.9	1
		3.02×10^{11}	30.7 ± 1.7	2
		1.54×10^{11}	39.0 ± 4.3	3
SEC	110 k EVs	fr 4: 2.29×10^{10}<br>fr 5: 8.06×10^{9}	46.6 ± 3.2<br>16.4 ± 2.8	1
		fr 4: 2.00×10^{10}<br>fr 5: 3.08×10^{9}	40.5 ± 3.4<br>31.2 ± 5.2	2
		fr 4: 2.82×10^{10}<br>fr 5: 2.48×10^{10}	57.3 ± 9.6<br>50.4 ± 10.0	3
	20 k EVs	fr 4: 3.35×10^{9}<br>fr 5: 3.38×10^{9}	17.0 ± 0.2<br>17.2 ± 1.9	1
		fr 4: 5.77×10^{8}<br>fr 5: 5.86×10^{9}	14.6 ± 1.2<br>29.7 ± 7.8	2
		fr 4: 1.23×10^{10}<br>fr 5: 7.71×10^{9}	24.9 ± 2.7<br>15.6 ± 4.0	3

Supplementary references

[1] M. Puhka *et al.*, “KeepEX, a simple dilution protocol for improving extracellular vesicle yields from urine,” *European Journal of Pharmaceutical Sciences*, vol. 98, pp. 30–39, Feb. 2017, doi: 10.1016/j.ejps.2016.10.021.

[2] C. Théry *et al.*, “Minimal information for studies of extracellular vesicles 2018 (MISEV2018): a position statement of the International Society for Extracellular Vesicles and update of the MISEV2014 guidelines,” *Journal of Extracellular Vesicles*, vol. 7, no. 1, Jan. 2018, doi: 10.1080/20013078.2018.1535750.
